# Loss of the Greatwall Kinase Weakens the Spindle Assembly Checkpoint

**DOI:** 10.1371/journal.pgen.1006310

**Published:** 2016-09-15

**Authors:** M. Kasim Diril, Xavier Bisteau, Mayumi Kitagawa, Matias J. Caldez, Sheena Wee, Jayantha Gunaratne, Sang Hyun Lee, Philipp Kaldis

**Affiliations:** 1 Institute of Molecular and Cell Biology (IMCB), A*STAR (Agency for Science, Technology and Research), Singapore, Republic of Singapore; 2 Program in Cancer and Stem Cell Biology, Duke-NUS Graduate Medical School, Singapore, Republic of Singapore; 3 Department of Biochemistry, National University of Singapore (NUS), Singapore, Republic of Singapore; 4 Department of Anatomy, National University of Singapore (NUS), Singapore, Republic of Singapore; Stowers Institute for Medical Research, UNITED STATES

## Abstract

The Greatwall kinase/Mastl is an essential gene that indirectly inhibits the phosphatase activity toward mitotic Cdk1 substrates. Here we show that although Mastl knockout (Mastl^NULL^) MEFs enter mitosis, they progress through mitosis without completing cytokinesis despite the presence of misaligned chromosomes, which causes chromosome segregation defects. Furthermore, we uncover the requirement of Mastl for robust spindle assembly checkpoint (SAC) maintenance since the duration of mitotic arrest caused by microtubule poisons in Mastl^NULL^ MEFs is shortened, which correlates with premature disappearance of the essential SAC protein Mad1 at the kinetochores. Notably, Mastl^NULL^ MEFs display reduced phosphorylation of a number of proteins in mitosis, which include the essential SAC kinase MPS1. We further demonstrate that Mastl is required for multi-site phosphorylation of MPS1 as well as robust MPS1 kinase activity in mitosis. In contrast, treatment of Mastl^NULL^ cells with the phosphatase inhibitor okadaic acid (OKA) rescues the defects in MPS1 kinase activity, mislocalization of phospho-MPS1 as well as Mad1 at the kinetochore, and premature SAC silencing. Moreover, using *in vitro* dephosphorylation assays, we demonstrate that Mastl promotes persistent MPS1 phosphorylation by inhibiting PP2A/B55-mediated MPS1 dephosphorylation rather than affecting Cdk1 kinase activity. Our findings establish a key regulatory function of the Greatwall kinase/Mastl->PP2A/B55 pathway in preventing premature SAC silencing.

## Author Summary

Cdk1 phosphorylates many substrates in mitosis and simultaneoulsy reduces the activity of the corresponding phosphatase PP2A through the Greatwall kinase/Mastl. When Mastl is deleted, cells progress through mitosis with missegregated chromosomes, which become unraveled. However, the molecular mechansims by which Mastl promotes proper chromosome segregation and mitotic progression remain elusive. In this study, we show that the Cdk1->Greatwall kinase/Mastl->PP2A pathway plays a central role in regulating the spindle assembly checkpoint (SAC) by preventing premature SAC silencing. We further demonstrate that Mastl is required for multi-site phosphorylation of the essntial SAC protein MPS1 as well as robust MPS1 kinase activity in mitosis by inhibiting PP2A/B55-mediated MPS1 dephosphorylation. Our findings establish the requirement of Mastl for robust SAC maintenance.

## Introduction

The activity of Cdk1/cyclin B is essential for cells to enter and complete mitosis. As recently shown in Xenopus and Drosophila, the phosphatase activity that dephosphorylates Cdk1 substrates is inhibited simultaneously with the peak of Cdk1 activity when cells enter mitosis to ensure maximal phosphorylation of Cdk1 substrates. Cdk1 phosphorylates and activates the Greatwall kinase/Mastl, which then phosphorylates Ensa or Arpp19 enabling them to bind and inhibit the phosphatase PP2A/B55 [1–4]. The Greatwall kinase is required for entry into mitosis in Xenopus [5] and similarly in human cells when Mastl was silenced completely [6], whereas mouse cells deleted for Mastl were reported to enter mitosis [7]. In contrast to mitotic entry, there is agreement that Mastl is important after nuclear envelope breakdown (NEBD) for exit from mitosis and cytokinesis [6–9]. In the context of Mastl deletion, the early mitotic defects have not been defined precisely and this is compounded by a lack of knowledge of specific PP2A substrates, which are dephosphorylated in the absence of Mastl. The only known target of Gwl/Mastl->PP2A is PRC1, an essential component assembling the central spindle during mitotic exit, with Thr481 being dephosphorylated by PP2A/B55 [8]. Therefore, identifying specific targets of the Greatwall kinase/Mastl->PP2A/B55 pathway is essential for understanding its *in vivo* functions.

One of the functions of Cdk1 is related to the spindle assembly checkpoint (SAC), which must be activated every time cells enter mitosis but needs to be silenced after all chromosomes have been properly attached to microtubules (for reviews see [10–12]). Nevertheless, the mechanism of silencing SAC, at a time where Cdk1 activity is still high, remains an open question. Recent studies elegantly demonstrated that besides cyclin B1 degradation, dephosphorylation of Cdk1 substrates is essential for regulation of the SAC and progression through anaphase [13–15]. Identifying the Cdk1-phosphorylated targets that need to be dephosphorylated to silence SAC is a major challenge, although the kinase MPS1 was suggested to be a potential candidate [13,16].

In this study, using conditional knockout mice for Mastl, we show the requirement of Mastl for robust spindle assembly checkpoint (SAC) maintenance. Using mass spectrometry, we have identified several mitotic targets of Cdk1 phosphorylation including MPS1 that are prematurely dephosphorylated in Mastl^NULL^ MEFs without altering the overall activity of Cdk1. Notably, we show that in MEFs lacking Mastl, mitotic multi-site phosphorylation of MPS1 as well as its kinase activity are compromised, which can be directly regulated by Cdk1 and PP2A/B55. Our findings reveal that the Cdk1->Greatwall kinase/Mastl->PP2A/B55 pathway controls mitotic phosphorylation of MPS1 and is essential for full kinase activity of MPS1 during mitosis, which may partly explain the requirement of Mastl for robust SAC maintenance.

## Results

### Absence of Mastl impairs embryonic development and cell proliferation due to abnormalities in mitosis

We generated a conditional (hereafter referred to as Mastl^FLOX^) knockout mouse model for the Mastl gene by inserting LoxP recombination sites on both sides of exon 4 in the mouse Mastl genomic locus [17]. In brief, the deletion of Mastl gene (hereafter referred to as Mastl^NULL^) was obtained by excision of the exon 4 from the control Mastl^FLOX^ allele using a constitutively expressed (β-actin-Cre), tissue specific (Albumin-Cre), or tamoxifen inducible [Rosa26-CreERT2 or Esr1 (CreERT2)] Cre recombinase. Germ line deletion of the Mastl gene resulted in embryonic lethality (S1A Fig). In depth investigation of various developmental stages in embryos revealed that early stage embryos (E3.5 blastocysts) were viable and succeeded to implant normally (S1B Fig). However, in comparison to control embryos, their growth was arrested before E7.5 and they did not develop further (S1B and S1C Fig). To exclude that functions of Mastl are limited to early embryonic development only, we utilized conditional knockout strategies in more advanced stage embryos and in different tissues in adult mice using the inducible Rosa26-CreERT2 (S1D and S1E Fig) as well as specifically in liver using Albumin-Cre (S1E and S4 Figs). Deletion of Mastl starting from E10.5 led also to embryonic lethality resulting in reduced cellularity in all organs analysed as well as haemorrhaging in the embryos (S1D Fig). Liver specific deletion of Mastl in hepatocytes induced abnormalities in their nuclear morphology (S1Ei and S1Eii Fig). Deletion of Mastl in adult mice by tamoxifen injection resulted in lethality within 7–8 days, accompanied with severe degeneration of the crypt morphology in the intestine (S1Eiii and S1Eiv Fig). The latter results were similar to what we observed in Cdk1^NULL^ mice [18]. These observations suggest that the Mastl kinase is essential for proliferation in adult organs and during the developmental stages we tested.

**Fig 1:**
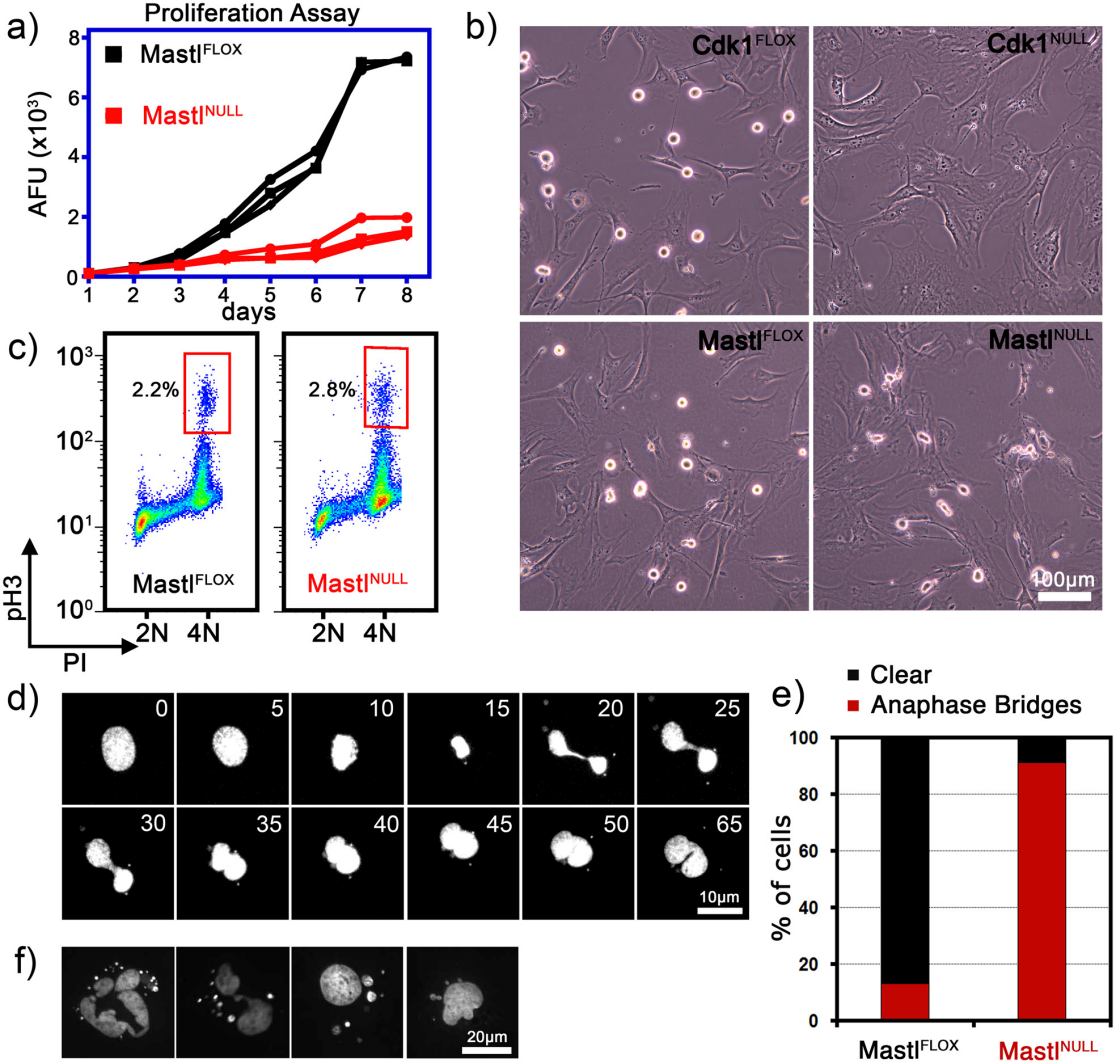
Growth analysis of Mastl^NULL^ MEFs. (A) Three MEF lines isolated from different embryos were treated with 4-OHT or DMSO to induce Mastl knockout (Mastl^NULL^) and their proliferative potential was monitored by Alamar Blue proliferation assays for 8 days. AFU, arbitrary fluorescence units. (B) MEFs were synchronized and recombination in the Mastl and Cdk1 loci were induced as described in the Methods section. Cells were arrested in mitosis for 4 hours using 5μM Eg5 inhibitor S-Trityl-L-cysteine (STLC) starting from 20 hours after release into full growth medium. Still pictures were taken using phase-contrast microscopy. Scale bar 100μm. (C) MEFs were fixed 24 hours after release into full growth medium and stained with anti-phospho-histone H3 Ser10 antibodies (pH3) to quantify mitotic cells using FACS analysis. (D) MEFs expressing the histone H2B-YFP fusion protein were analyzed by time-lapse microscopy. Still images of a dividing Mastl^NULL^ cell were acquired every 5 minutes. Scale bar 10μm. (E) Quantification of appearance of anaphase bridges in Mastl^FLOX^ and Mastl^NULL^ MEFs expressing the H2B-YFP. (F) Mastl^NULL^ cells were fixed and stained with DAPI 72 hours after synchronization and release into full growth medium. Scale bar 20μm.

To investigate whether Mastl is required for cell proliferation *in vitro*, we isolated Mastl^FLOX/FLOX^ mouse embryonic fibroblasts (MEFs) carrying the Esr1 (CreERT2) transgene. Induction of Cre-mediated recombination in the Mastl locus by addition of 4-hydroxytamoxifen (4-OHT) resulted in the loss of the Mastl mRNA and protein (S2A Fig). Primary Mastl^NULL^ MEFs were unable to proliferate (Fig 1A) and displayed multi-lobular nuclear morphology (Fig 1D and 1F, S2B Fig). Further analysis of the cell cycle profile of synchronized MEFs by FACS indicated that although they progressed through S phase, Mastl^NULL^ MEFs displayed an increased G2/M population, as well as polyploidy and cell death (S2C Fig). Because of the increased G2/M population in absence of Mastl, we investigated whether these cells can enter mitosis. Unlike Cdk1^NULL^ [18], Mastl^NULL^ MEFs did enter mitosis albeit with a delay (Fig 1B and 1C). However, time-lapse microscopy of Mastl^NULL^ MEFs stably expressing histone H2B-YFP (chromosome marker) revealed that these cells progressed through mitosis despite the presence of misaligned chromosomes (Fig 1D, S1 and S2 Movies) but never completed cytokinesis. Quantification from time-lapse images indicated that more than 90% of Mastl^NULL^ MEFs progressed through mitosis with anaphase bridges whereas ~10% of control Mastl^FLOX^ MEFs did so (Fig 1E). This mitotic phenotype with anaphase bridges resulted in binucleated cells (Fig 1D) or rupture of the nucleus bearing micronuclei in Mastl^NULL^ MEFs (Fig 1F).

Biochemical comparison of synchronized Mastl^FLOX^ and Mastl^NULL^ MEFs indicated comparable expression levels of cyclins, Cdks, and inhibitory phosphorylation of Cdk1 on Y15 (S3A Fig). Furthermore, kinase activity associated with immunoprecipitated Cdk1 and cyclin B1 was not decreased in Mastl^NULL^ compared to Mastl^FLOX^ MEFs. In contrast, Cdk2 and cyclin A2 associated kinase activity was slightly reduced in Mastl^NULL^ MEFs (S3B and S3C Fig). These results suggest that the observed accumulation in G2/M is unlikely due to a decrease in Cdk1 kinase activity.

To further confirm our results in an *in vivo* model of cell division, we deleted Mastl specifically in hepatocytes using Albumin-Cre and performed partial hepatectomy (PHx) in these mice (S4 Fig). Similar to Cdk1 [18] and cyclin A2 (S4D Fig), the expression of the Mastl protein increased between 24 and 72 hours after PHx in Mastl^FLOX^ mice, but not in Mastl^NULL^ liver at 48 hours after PHx, as expected (S4D and S4E Fig). Analysis of histological sections taken 48 hours post PHx displayed an elevated mitotic index as judged by phopho-histone H3 staining (S4B Fig). Mastl^NULL^ hepatocytes were unable to divide properly and displayed frequently binucleated morphology as well as anaphase bridges (S4A–S4C Fig). The phenotype of Mastl^NULL^ observed *in vivo* (hepatocytes) is reminiscent of Mastl^NULL^ MEFs (see Fig 1), indicating that completely different cell types react similarly to the deletion of Mastl.

### Proper progression through mitosis requires Mastl

**Fig 2:**
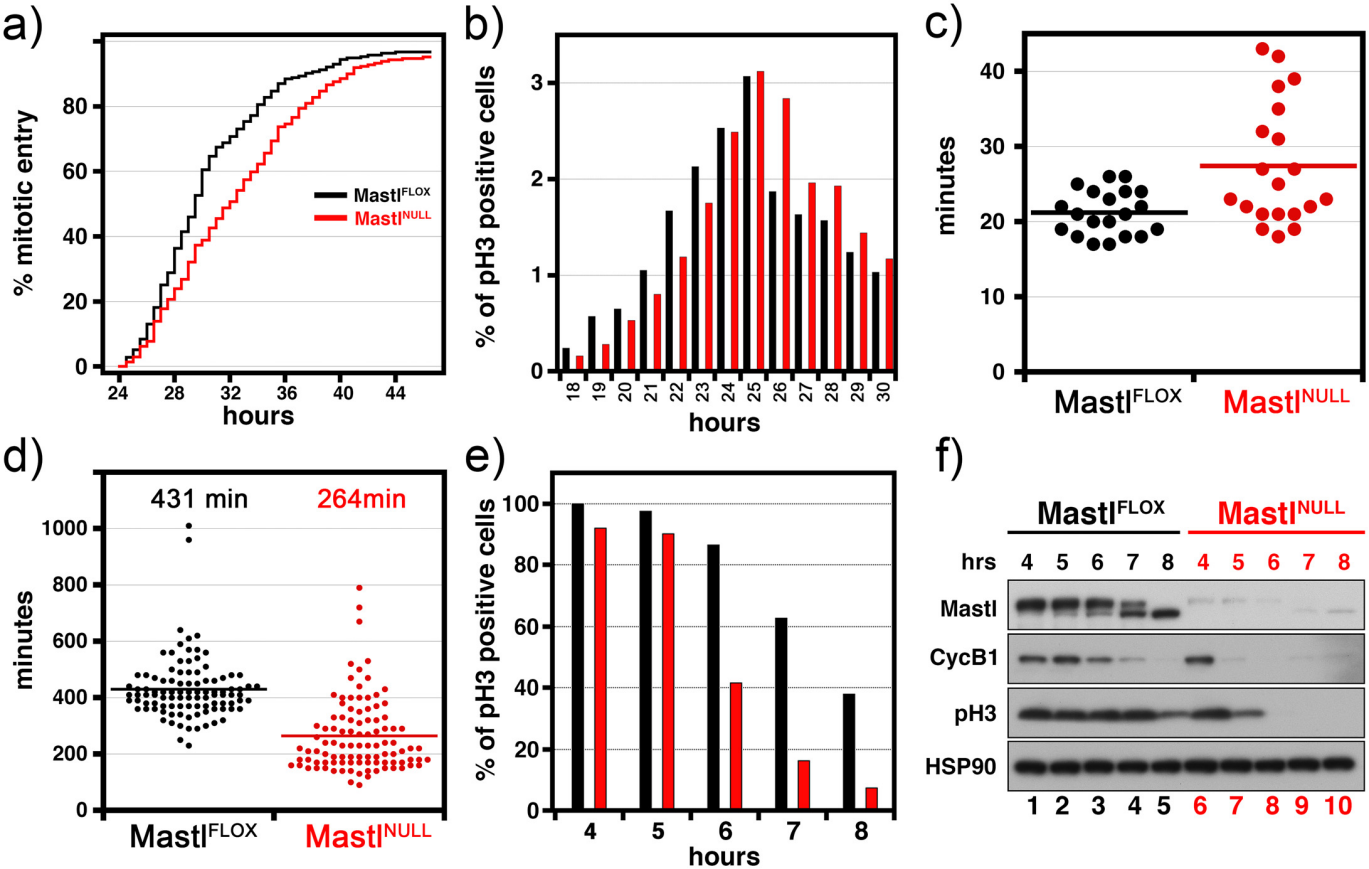
Mitotic analysis of Mastl^NULL^ MEFs. (A) Immortalized MEFs expressing histone H2B-YFP were synchronously released to enter the cell cycle and monitored by time-lapse microscopy. Images were taken every 5 minutes, starting from 23 hours after release. The mitotic entry time-point was determined from rounding up of the cell body and chromosome condensation (N>200). (B) Primary MEFs were synchronously released to enter the cell cycle and collected at different time-points after release to determine the mitotic index (the percentage of cell population in mitosis) by FACS analysis using phospho-histone H3 Ser10 (pH3) staining. (C) Immortalized MEFs expressing pCSII-EF-mAG-hGeminin were synchronized, induced for Mastl deletion (Mastl^NULL^) and released into complete medium (see Methods). 24 hours after serum release, cells were monitored under the microscope by time-lapse imaging. Mitotic duration was calculated as the time it takes from nuclear envelope breakdown (beginning of mitosis) to degradation of the Geminin protein (end of mitosis). *p*<0.001 according to Student’s t-test. (D) Immortalized MEFs expressing H2B-YFP were synchronously released to enter the cell cycle and were treated with 500ng/ml nocodazole starting from 24 hours after release and were monitored by time-lapse microscopy. Mitotic duration for mitotic slippage was calculated as the time it takes from nuclear envelope breakdown (beginning of mitosis) to chromosome decondensation (N = 100, *p*<0.0001 Student’s *t*-test). (E-F) Primary MEFs were synchronously released to enter the cell cycle, were treated with 500ng/ml of nocodazole starting from 20 hours. Cells were collected by pipetting (mitotic shake-off), further incubated in the presence of nocodazole and (E) fixed at different time points to determine the percentage of cells with phospho-histone H3 (pH3) on Ser10 by FACS analysis or (F) lysed for immunoblot analysis using the indicated antibodies.

Quantitatively measuring the entry and the length of mitosis using time-lapse video microscopy analysis revealed a slight but measurable delay in mitotic entry in unperturbed Mastl^NULL^ MEFs but despite this, the majority of cells entered mitosis (Fig 2A). This delay was further verified by quantification of mitotic cells by FACS analysis (Fig 2B). Moreover, in unperturbed cells, the duration of mitosis was somewhat prolonged in Mastl^NULL^ MEFs (Fig 2C) most likely due to poorly aligned chromosomes at the metaphase plate (S1 and S2 Movies). Although this delay in mitotic entry was one of three major phenotypes in Mastl^NULL^ MEFs; i) mitotic entry defects, ii) premature SAC silencing [see below], and iii) cytokinesis defects [8]; it will not be further investigated in this manuscript. Nonetheless, once Mastl^NULL^ MEFs entered mitosis, surprisingly they progressed through mitosis in the presence of chromosome segregation defects (Fig 1D–1F, S1 and S2 Movies) but never completed cytokinesis properly, which resulted in accumulation of Mastl^NULL^ MEFs with ≥4N DNA content (S2C Fig).

Under normal conditions, robust SAC signalling ensures mitotic arrest until these attachment errors are properly corrected [10]. To address whether this premature anaphase onset with misaligned chromosomes was due to weakened SAC signalling, MEFs were treated with microtubule poisons including the microtubule depolymerizer nocodazole and the percentage of cells arrested in mitosis was analysed by FACS. Under these conditions, cells are arrested in a prometaphase-like state until eventually cyclin B1 degradation and subsequent chromosome decondensation without separation takes place; a process called mitotic slippage [19]. Time-lapse microscopy analysis indicated that in nocodazole treated Mastl^NULL^ MEFs the mitotic duration was reduced compared to control Mastl^FLOX^ MEFs (Fig 2D). Furthermore, analysis of the percentage of cells remaining in mitosis measured as positive for phospho-histone H3 on Ser10 (pH3) staining using FACS confirmed that nocodazole treated Mastl^NULL^ MEFs exited mitosis significantly faster than control cells (Fig 2E, S5A Fig). Likewise, immunoblotting of whole cell lysates of these mitotic cells indicate a reduction of the pH3 level and that the degradation of cyclin B1 was accelerated in absence of Mastl in comparison to control cells (Fig 2F). This phenotype of mitotic slippage suggests that Mastl is required for robust and persistent SAC signalling.

### Phosphorylation of MPS1 on Ser820 is regulated by both Cdk1 and PP2A

**Fig 3:**
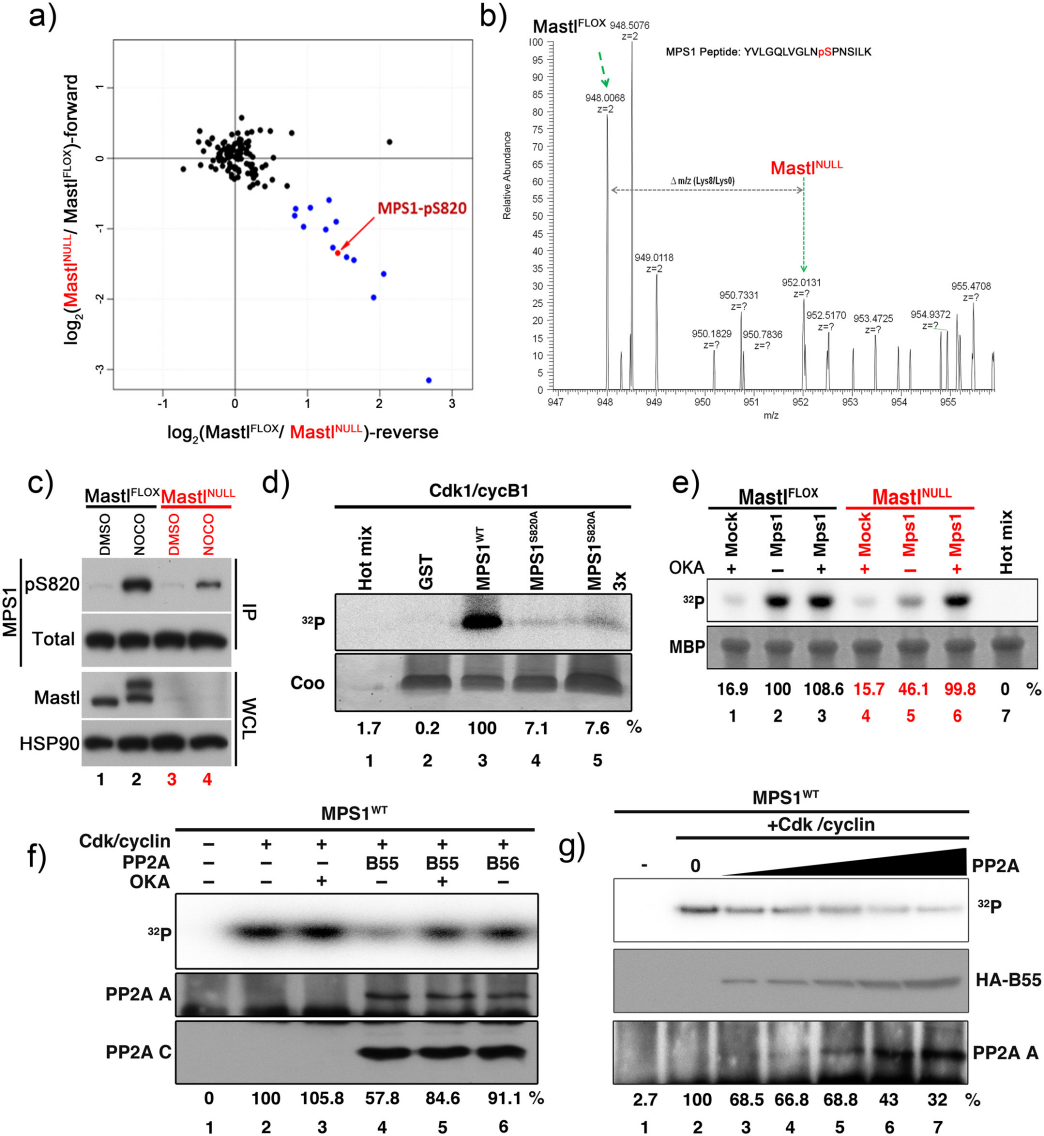
Mastl is required for phosphorylation and full activity of MPS1. (A) Phospho-proteomic analysis of mitotic arrested MEFs was performed using SILAC. The log2 SILAC ratio of phospho-peptides in each mass spectrometry run is plotted as the forward experiment against the reverse experiment. Data points colored in blue denote down-regulated phosphorylation sites in Mastl^NULL^ cells with at least 1.5 fold change. Black points indicate peptides with unchanged phosphorylation status. (B) MPS1 S820 phospho-peptide MS spectrum: the intensities of SILAC peptide pairs (heavy signal corresponding to Mastl^NULL^ cells and light signal to Mastl^FLOX^) are shown. (C) Immortalized MEFs expressing lentivirally transduced HA-tagged MPS1 (human) were synchronized and released as indicated in Fig 2D. Cells were treated with or without 500ng/ml nocodazole between hours 24–28 after release. Whole cell lysate (WCL) for each condition were prepared and subjected to immunoblot analysis for Mastl and HSP90. HA-MPS1 was immunoprecipitated (IP) using anti-HA antibodies and probed for phosphorylated or total MPS1. (D, F, G) GST-tagged recombinant wild type (WT) or non-phosphorylatable mutant (S820A) MPS1 peptide fusion protein was purified and subjected to *in vitro* kinase assay with recombinant Cdk1/cyclin B1 complexes (D; see Methods; MPS1^S820A^ 3x designated an amount of three times of the substrates from lane 4). For F and G, phosphorylated GST-MPS1^WT^ peptide fusion protein was subjected to *in vitro* phosphatase assay using immunoprecipitated HA-tagged B55 or B56 complexes in presence (+) or absence (-) of 200nM OKA. A representative image for each experiment is shown and representative results for quantification of ^32^P intensity signals of phosphorylated GST-MPS1 peptide fusion protein are shown as percentage derived from lane 6 for D, lane 2 for F-G. For F-G, levels of the PP2A A scaffold and PP2A C catalytic subunits co-immunoprecipitated are shown. For G, *in vitro* phosphatase assay was performed using immunoprecipitated PP2A complexes from increasing amounts of whole cell lysates from HA-B55 overexpressing 293T cells (25, 50, 100, 250 and 500μg) and phosphorylated GST-MPS1 peptide fusing protein as a substrate. Western blots for HA-B55 (middle panel) and the PP2A A scaffold subunit (bottom panel) are shown. (E) Immortalized MEFs synchronously released into full growth medium were transfected with plasmids encoding Myc-tagged MPS1 (human). Cells were arrested in mitosis by treating with 500ng/ml nocodazole for 4 hours between hours 24–28 after serum release. Cells were additionally treated with 200nM OKA (+) or DMSO (-) between hours 27–28 as indicated. Myc-MPS1 was immunoprecipitated using anti-Myc antibodies and subjected to *in vitro* kinase assay using 5μg MBP as a substrate and recombinant Cdk1/cyclin B1 as kinase (see Methods). A representative experiment is shown and representative results for quantification of ^32^P intensity signals of phosphorylated MBP are shown as percentage derived from lane 2.

Since numerous proteins are involved in the SAC (for a discussion see [10]), we aimed to identify specific proteins whose phosphorylation status is changed when Mastl is knocked out and therefore employed mass spectrometry-based quantitative phospho-proteomic analysis. To achieve this, we collected mitotically arrested Mastl^FLOX^ and Mastl^NULL^ MEFs cultured in SILAC media and compared their phospho-proteomes. Analysis of each sample was performed in duplicate and by reversing the isotope labelling between conditions in the second replicate (for detailed information see Materials and Methods). Our results indicated that while 136 of the identified phosphorylation sites did not change their phosphorylation level (Fig 3A, black dots), 14 phosphorylation sites corresponding to 12 different proteins were specifically decreased in Mastl^NULL^ cells with at least 1.5 fold-change (Fig 3A, blue dots and S1 Table), consistent with Mastl regulating either directly or indirectly a phosphatase activity. This “mini” screen was not saturating for technical reasons but among the proteins with decreased phosphorylation status, we identified TTK [20], a dual specificity kinase also known as MPS1 [21]. We identified serine 820 (821 in Human) of MPS1 as the site with decreased phosphorylation (Fig 3B). Since phosphorylation of MPS1 is required for SAC signalling and proper chromosome segregation and its phosphorylation on S820 peaks during mitosis [16,22–28], we decided to focus our analysis on the regulation of MPS1 activity in SAC signalling. To connect the functions of MPS1 to the observed phenotype in Mastl^NULL^, we treated nocodazole-arrested Mastl^FLOX^ MEFs with the MPS1 inhibitor reversine [29] (S5B Fig, blue bars). This led to rapid mitotic slippage similar to what we have observed in Mastl^NULL^ MEFs, indicating that MPS1 could be responsible in principle for some of the phenotypes observed in the absence of Mastl.

The regulation of MPS1 activity and its phosphorylation is complex and not fully understood [16,22,30,31]. For example, T675 and T685 have been suggested to be autophosphorylation sites essential for MPS1 activity, while S820 is more likely phosphorylated by other kinases to regulate its localization [16,24]. To determine whether MPS1 functions are deregulated in Mastl^NULL^ MEFs, immunoblot analysis using phospho-specific antibodies against MPS1 were performed. In Mastl^NULL^ MEFs harvested by mitotic shake-off after nocodazole treatment, the levels of phosphorylated S820 was markedly reduced in comparison to Mastl^FLOX^ MEFs (Fig 3C). To determine whether S820 could be phosphorylated directly by Cdk1, an *in vitro* kinase assay was performed using recombinant Cdk1/cyclinB1 complexes and a GST-MPS1 fusion protein as substrate. In order to express this fusion protein in bacteria, and simultaneously avoid interfering signals from the numerous potential phosphorylation sites of MPS1 including autophosphorylation, we fused a short peptide of MPS1 (residues 811–831) containing S820 downstream of a GST tag. Cdk1/cyclin B1 phosphorylated S820 but not the non-phosphorylatable mutant S820A (Fig 3D) as expected [16]. In contrast to Cdk1, Mastl was not able to directly phosphorylate S820 *in vitro* (S6B Fig). Together, these results confirm that Cdk1 phosphorylates MPS1 [16] and agree with the increased phosphorylation of S820 in nocodazole treated cells (Fig 3C, lanes 1 & 4), although other kinases including MAPK may also phosphorylate MPS1 at S820 or other sites. Since there is no clear consensus on how the different phosphorylation sites in MPS1 affect its kinase activity, we aimed to measure the change in total MPS1 kinase activity directly. To achieve this, we immunoprecipitated MPS1 and determined its kinase activity towards the substrate myelin basic protein (MBP). MPS1 kinase activity was substantially reduced in mitotic extracts from Mastl^NULL^ MEFs compared to Mastl^FLOX^ MEFs (Fig 3E, compare lanes 2 & 5). Since Mastl regulates the PP2A phosphatase activity through ENSA/Arpp19 [1–3], we evaluated whether the decrease in MPS1 activity was due to the increase in PP2A activity in Mastl^NULL^ MEFs. Indeed, mitotic extracts from Mastl^NULL^ MEFs treated with OKA restored MPS1 activity similar to the levels seen in Mastl^FLOX^ MEFs (Fig 3E, lane 6 and S6A Fig, lane 6). Consistent with this result, Mastl^NULL^ and Mastl^FLOX^ MEFs when treated with OKA display a similar mitotic slippage rate (S5C Fig). Although we have already shown that Cdk1 activity was not changed in absence of Mastl (S3B and S3C Fig), we further tested whether ectopic expression of a non-degradable form of cyclin B1 (Δ85cyclin B1), which keeps Cdk1 activity high [32], would restore MPS1 activity (S6C Fig). Extracts from Mastl^FLOX^ MEFs expressing Δ85cyclin B1 displayed four-fold increased MPS1 activity due to accumulation of cells in mitosis but there was no additional increase in Mastl^NULL^ cells (S6C Fig), indicating that increasing Cdk1 activity does not elevate MPS1 activity in Mastl^NULL^. Together, our data reveal the requirement of Mastl for full MPS1 kinase activity and persistent SAC signalling in mitosis. Furthermore, our data also suggest that the decreased activity of MPS1 is not due to a decrease in Cdk1 kinase activity, but an increase in the PP2A phosphatase activity toward MPS1 in Mastl^NULL^ MEFs.

Despite the rescue of MPS1 activity and phosphorylation in Mastl^NULL^ cells by OKA treatment, this could be an indirect consequence of PP2A inhibition. To test whether S820 MPS1 is dephosphorylated directly by PP2A as well as to identify the PP2A regulatory B subunit responsible for this dephosphorylation, we performed *in vitro* phosphatase assay using immunopurified PP2A/B55α or PP2A/B56α complexes. Incubation of phosphorylated pS820 MPS1 with immunoprecipitated PP2A/B55α resulted in a marked dephosphorylation of S820 MPS1 (Fig 3F, lane 4), but not in the presence of OKA (lane 5). Although both PP2A/B55 or PP2A/B56 complexes displayed phosphatase activity towards phosphorylated Rb (S6D Fig), the dephosphorylation of S820 MPS1 was more specific for B55α because immunoprecipitated PP2A/B56α was not as potent as PP2A/B55α (Fig 3F, lane 6). Of note, this difference was not simply due to formation and purification of the PP2A holocomplex as determined by immunoblot analysis, indicating similar amounts of the PP2A A and C subunits co-purified with B55α and B56α (Fig 3F, bottom panels). Furthermore, incubation with immunorecipitated PP2A complexes from increasing amounts of whole cell lysates of HA-B55/B56 overexpressing 293T cells caused a concentration-dependent dephosphorylation of S820 MPS1 (Fig 3G).

Together, these results indicate that PP2A/B55 dephosphoylates S820 MPS1 *in vitro* and that the Greatwall kinase/Mastl->PP2A/B55 pathway may be directly responsible for preventing premature dephosphorylation of at least S820 MPS1 in mitosis.

To exclude any effects of deletion of Mastl on the expression of PP2A, Arpp19, and ENSA, we further analysed the mRNA expression of any of the key PP2A subunits and no significant changes were observed between Mastl^FLOX^ and Mastl^NULL^ MEFs for the different isoforms of the regulatory PP2A subunits B55 and B56 (S7 Fig). While the mRNA expression level of Arpp19 was low or undetectable in primary MEFs by qPCR (Ct value 32.9), no significant change in ENSA mRNA expression level was observed between Mastl^NULL^ and Mastl^FLOX^ MEFs at each time point (S7 Fig). Nonetheless, we detected an increase of ENSA at protein level in Mastl^NULL^ MEFs in comparison to the control Mastl^FLOX^ cells (S3A Fig). Due to the lack of appropriate reagents, we were unable to determine the decrease in ENSA pS67 phosphorylation in Mastl^NULL^ MEFs similar as also in a recent study [7] on Greatwall kinase/ Mastl knockout mice.

**Fig 4:**
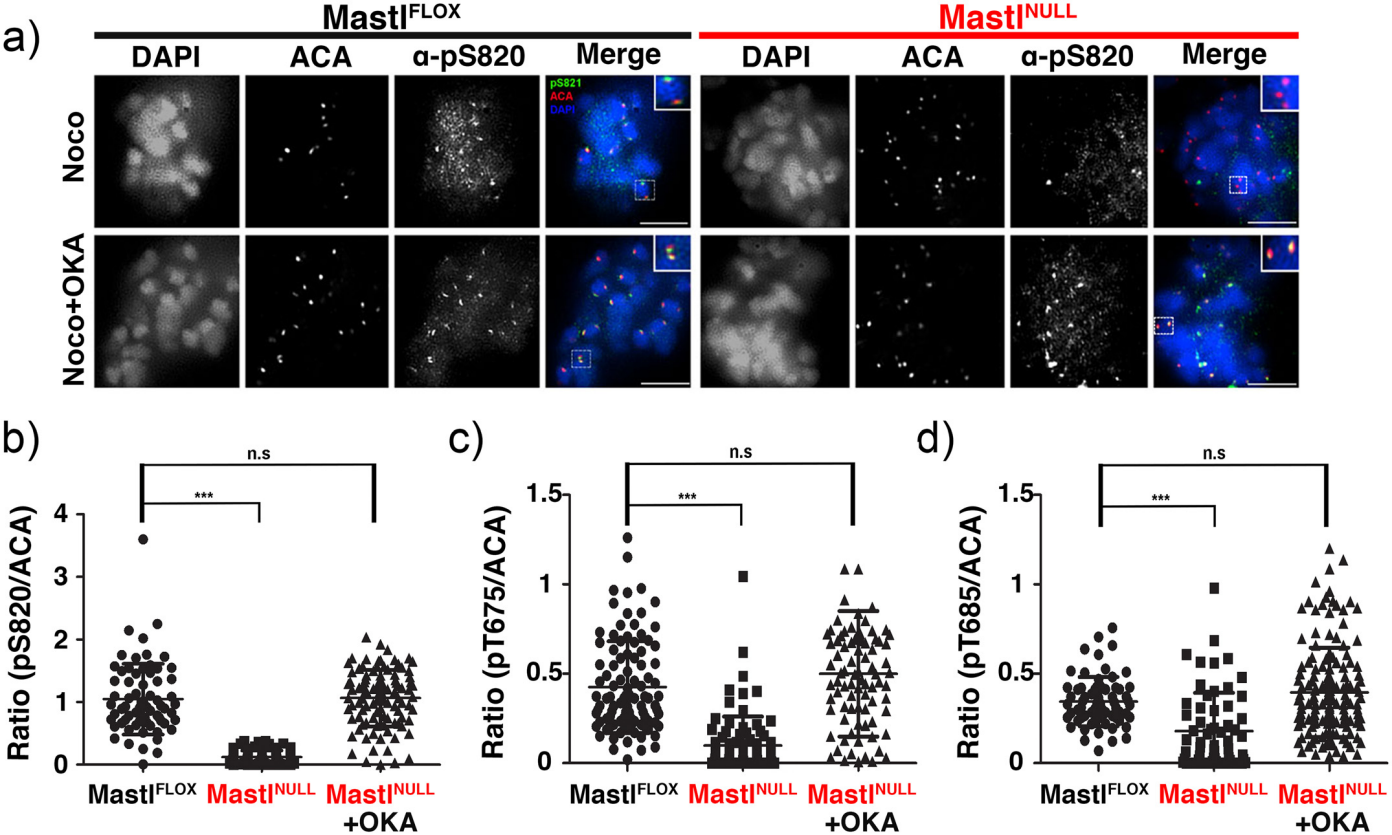
Kinetochore localization of MPS1 phosphorylated on S820. Primary MEFs were synchronized and released to enter the cell cycle as in Fig 2C. Cells were treated with 500ng/ml nocodazole for 4 hours between hours 20–24 after serum release. Where indicated, cells were treated with 200nM OKA for one hour to inhibit PP2A activity between hours 23–24 after serum release. Cells were fixed with PFA or ice-cold MeOH and subjected to immunofluorescence analysis using ACA and antibodies against MPS1-pS820. Insets represent the boxed areas. Scale bar 5 μm. Quantification of kinetochore-localized MPS1-pS820 (B), MPS1-pS675 (C), MPS1-pS685 (D) in nocodazole arrested Mastl^FLOX^ and Mastl^NULL^ cells (N>10 cells per each condition, +/- standard deviation). ACA stained kinetochores were randomly selected with the NIS-Elements AR software using automated measurement and relative kinetochore intensity of phospho-MPS1 to ACA was determined. To determine the statistical significance, a Student’s *t*-test was performed. (n.s. not significance; ***p<0.0001, Student’s *t*-test, unpaired).

To link the activity of MPS1 to its kinetochore function, we analyzed the localization of the phosphorylated form of MPS1 using phospho-specific antibodies [24]. We were able to detect S820-phosphorylated MPS1 at the kinetochore of Mastl^FLOX^ MEFs treated with nocodazole, whereas it was significantly decreased or absent in Mastl^NULL^ MEFs (Fig 4A and 4B). Similar results were obtained using phospho-specific antibodies for MPS1 T675 and T685 (S8 Fig, Fig 4C and 4D), suggesting that Mastl is essential for regulating multi-site phosphorylation of MPS1 *in vivo*. Since Mastl is known to regulate PP2A activity through phosphorylation of ENSA or Arpp19 [2–4] and inhibition of PP2A activity by OKA rescued MPS1 activity from Mastl^NULL^ MEFs (see Fig 3E), we evaluated the ability of OKA to rescue the kinetochore localization of phosphorylated MPS1. Indeed, in Mastl^NULL^ MEFs treated with OKA, MPS1 phosphorylated on S820, T675, and T685 was restored correctly at the kinetochores similar to that seen in Mastl^FLOX^ MEFs (Fig 4A–4D, S8 Fig).

### Maintenance but not activation of the SAC is impaired in the absence of Mastl

The kinase activity of MPS1 is required for correct targeting and activation of a number of SAC regulators at the kinetochores [33] as cells enter mitosis. Notably, the relatively short duration of mitosis in Mastl^NULL^ MEFs upon treatment of microtubule poisons suggests SAC signalling defects (Fig 2D). However, in unperturbed mitosis, the duration of mitosis in Mastl^NULL^ MEFs was prolonged with poorly aligned chromosomes at the metaphase plate (Fig 2C, S1 and S2 Movies), suggesting that SAC signalling may be properly activated and established during mitotic entry but cannot be maintained robustly through mitosis.

**Fig 5:**
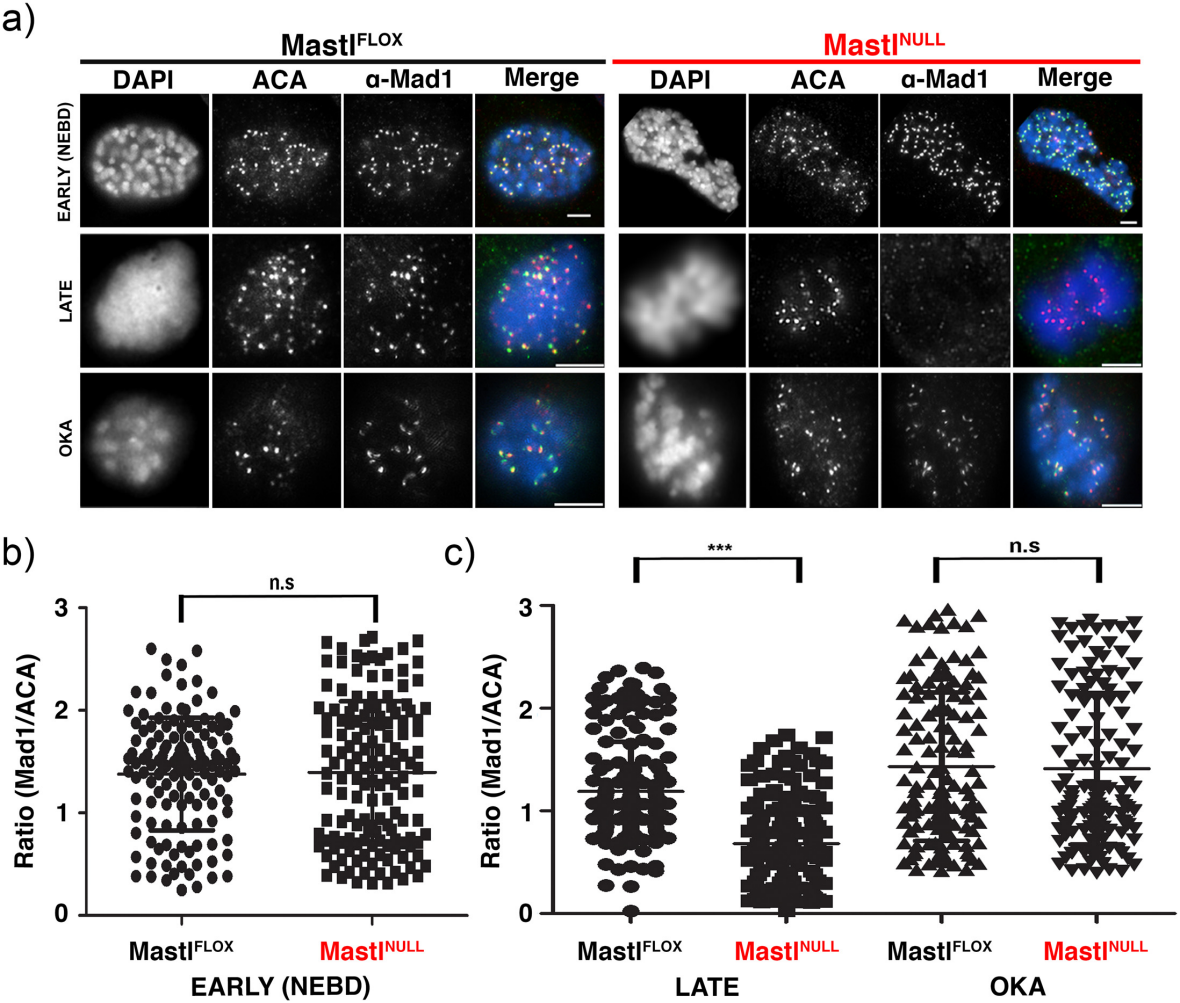
Kinetochore localization of Mad1 in MEFs in early and late mitosis. (A) Primary MEFs were treated as in Fig 4 to induce mitotic arrest and were fixed. Where indicated, cells were treated with 200nM OKA for one hour before fixation. Immunofluorescence analysis was performed using ACA and antibodies against Mad1. Different mitotic phases were determined with early mitotic cells determined by lightly condensed chromosomes that were scattered in the cytoplasm just after NEBD and late mitotic cells displaying a highly condensed chromosome mass typically caused by nocodazole treatment. Insets represent the boxed areas. Scale bar 5 μm. Quantification of kinetochore-localized Mad1 in Mastl^FLOX^ and Mastl^NULL^ cells in early prophase (NEBD) (B) or late prometaphase-like state by nocodazole treatment (C) [N>20 cells per each condition, ± standard deviation] as described in Fig 3B except using kinetochore-localized Mad1. To determine the statistical significance, a Student’s *t*-test was performed. (n.s. not significance; ***p<0.0001, Student’s *t*-test, unpaired).

To test this hypothesis, the kinetochore localization of the essential SAC protein Mad1 in early prophase (with lightly condensed chromosomes that were scattered in the cytoplasm just after NEBD) was determined by using immunofluorescence analysis using the centromeric marker ACA, as shown previously [30,31]. Notably, the kinetochore localization of Mad1 between Mastl^FLOX^ and Mastl^NULL^ MEFs displayed no significant difference in early prophase (Fig 5A (top panels) and Fig 5B), suggesting the correct establishment of proper SAC signalling in the absence of Mastl. In contrast, the kinetochore localization of Mad1 at late prometaphase-like stage (with a highly condensed chromosome mass typically caused by nocodazole treatment) was markedly defective in Mastl^NULL^ but not in Mastl^FLOX^ MEFs (Fig 5A (middle panels) and Fig 5C). Notably, OKA treatment efficiently restored the kinetochore localization of Mad1 at late prometaphase-like stage in Mastl^NULL^ MEFs similar to that seen in Mastl^FLOX^ MEFs (Fig 5A (bottom panels) and Fig 5C). Together, these results suggest that SAC signalling in Mastl^NULL^ MEFs is properly activated and established at the entry of mitosis, but it is unable to be maintained persistently even with misaligned chromosomes during progression through mitosis. Moreover, the defects in the kinetochore localization of phospho-MPS1 (T675, T685, S820) at late prometaphase-like stage in Mastl^NULL^ MEFs (see Fig 4 and S8 Fig) could not be detected in early prophase just after NEBD (S9A–S9C Fig). Thus, our results indicate that although the Cdk1->Greatwall kinase/Mastl->PP2A/B55 pathway may be responsible for MPS1 phosphorylation and full kinase activity at the kinetochores to maintain robust SAC signalling until completion of mitosis but not for the establishment and activation of SAC when cells enter mitosis. Taken together, we conclude that Mastl is important for robust SAC maintenance until the satisfaction of the checkpoint by bi-stably attaching microtubules to all kinetochores.

## Discussion

Here we show that the absence of the Greatwall kinase/Mastl abbrogates cell proliferation impairing embryonic development and tissue renewal in mice. Although it had been observed that Mastl deficient cells from Xenopus or human cell lines do not enter mitosis efficiently [5,6,9], our results revealed that genetic deletion of Mastl only delays mitosis entry as has been shown recently [7]. Mastl deficient unperturbed MEFs display a prolonged mitosis. However, once Mastl^NULL^ MEFs entered mitosis, a majority of cells prematurely progressed through mitosis with missegregated chromosomes, resulting in anaphase bridges and the generation of binucleated cells without completing cytokinesis. Furthermore, when treated with microtubule poisons to disrupt chromosome alignment and to activate the SAC, Mastl^NULL^ MEFs underwent mitotic slippage significantly faster than Mastl^FLOX^ MEFs. Concurrently, we observed decreases in phosphorylation levels and activity of MPS1, which coincided with the defective kinetochore localization of Mad1 and phosphorylated MPS1 in Mastl^NULL^ MEFs. Together, these results suggest defects in SAC signaling in Mastl^NULL^ MEFs. Notably, these defects occurred without decreasing the overall activity of Cdk1 in Mastl^NULL^ MEFs and they were efficiently rescued by inhbition of PP2A with OKA. Furthermore, we demonstrated that Cdk1 phospohrylation of MPS1 S820 can be reversed directly by PP2A/B55 *in vitro*. Collectively, our results indicate that the Cdk1->Greatwall kinase/Mastl->PP2A pathway plays a central role in the regulation of SAC, part of which is likely contributed by Mastl preventing PP2A/B55-mediated dephosphorylation of MPS1 in mitosis, although it is well possible that there are other substrates that regulate SAC since our “mini” screen was not saturating.

**Fig 6:**
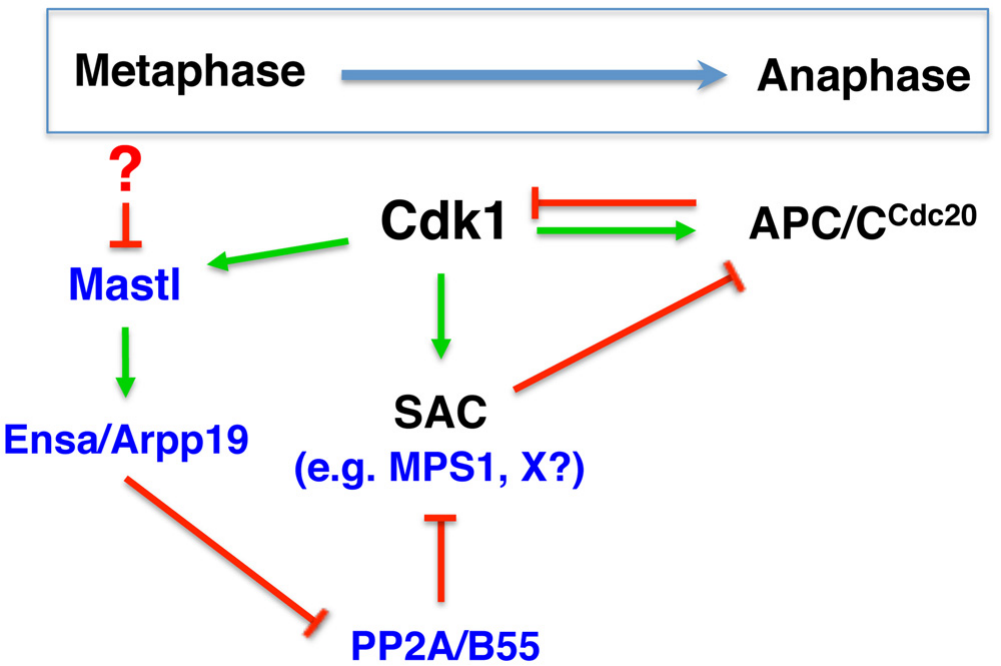
Model of SAC regulation by the Greatwall /Mastl kinase. Triangular regulation that controls the meta-to-anaphase transition whereby Cdk1 activates APC/C^Cdc20^ at the same time as it does SAC, which in turn inhibits APC/C^Cdc20^. Once APC/C^Cdc20^ is active, it will degrade cyclin B leading to inactivation of Cdk1 but this can only happen after SAC is silenced. Here we show that Mastl through PP2A/B55 regulates MPS1 and potentially other unidentified substrates (X) to control SAC activity. These intricate feedback loops may help to fine-tune the timing of meta-to-anaphase transition. How Mastl activity decreases during this transition to allow PP2A activity remains unknown, but this could involve phosphatases. Green and red colors indicate a positive and negative regulation, respectively.

The role of Cdk1 in promoting SAC signalling has been well documented [13,14,34], but we are the first to report the essential role of Mastl in robust SAC maintenance. Notably, we found that neither the activity of Cdk1 nor its inhibitory phosphorylation on Y15 was affected in absence of Mastl. To confirm this, we used non-degradable cyclin B1 to ensure Cdk1 fully active in mitosis but there was no increase in MPS1 activity in Mastl^NULL^ cells. These observations make it unlikely that Mastl affects Cdk1 activity though we cannot entirely exclude this possibility. The conundrum to be resolved is that Cdk1 activates APC/C^Cdc20^ at the same time as the SAC, which inhibits APC/C^Cdc20^ [13]. This triangle can only be resolved when Cdk1 activity drops, which simultaneously requires active APC/C^Cdc20^ (by degrading cyclin B) and silencing of the SAC (Fig 6). Our work suggests an additional regulatory loop, which includes Mastl, PP2A/B55, and MPS1, could contribute to the regulation of SAC silencing during mitotic progression. We propose that this regulatory loop allows SAC signalling to be robustly maintained by restraining PP2A, which removes activating phosphorylation of SAC components (e.g. MPS1 and maybe others), before all chromosomes are bi-stably attached. Although the regulation of Mastl activity has not been completely elucidated, inhibiting Mastl activity (such as by additional phosphatases) may also enable the SAC to be silenced even at a time when Cdk1 activity is still high and thereby activating APC/C^Cdc20^ (e.g. mitotic slippage). This is an attractive hypothesis to resolve the Cdk1->APC/C^Cdc20^->SAC conundrum.

The regulation of MPS1 including its kinetochore recruitment is complex and not fully understood [16,22,30,31]. MPS1 is phosphorylated on several sites during mitosis and in a Cdk1-dependent fashion, which may regulate its activity and/or its kinetochore localization [16,22,23,35,36]. Among these, phosphorylation sites T675 and T685 (T676 and T686 in human) regulate MPS1 activity by autophosphorylation [22–24,35]. Consistent with our observations that Cdk1/cyclin B and PP2A/B55 control the phosphorylation status of MPS1 on S820 during mitosis, previous phospho-proteome datasets of mitotic cells display a peak of phosphorylation of MPS1 on S820 during the mitosis [26–28]. However, it has been observed that phosphorylation on S820 is not essential for MPS1 activity *in vitro* [16,24] whereas our mass spectrometry analysis identified S820 phosphorylation as decreased in Mastl^NULL^ MEFs and therefore likely to be involved in MPS1 activity *in vivo*. Interestingly, a recent study by McCloy and colleagues reports that MPS1 phosphorylation on S820 as well as on S281 and S321 dramatically decrease during early mitosis in the presence of Cdk1 inhibitors [36]. Similar to the phenotype observed in Mastl^NULL^ MEFs, the decreased phosphorylation level of MPS1 leads to a drop of MPS1 activity and to the silencing of the SAC due by the dephosphorylation of MPS1 substrates [36].

Although phosphorylation of other MPS1 residues (e.g. T675, Fig 4A) may be also altered in Mastl^NULL^ MEFs since our mass spectrometry analysis was not saturating, our data indicate the potential role of S820 phosphorylation for MPS1 function. Indeed, reconstitution of a phospho-mimetic mutant for this residue in MPS1-depleted frog extracts rescued the kinetochore localization of MPS1 and SAC signalling, while non-phosphorylatable mutant S820A failed to do so [25]. We have tried to perform similar experiments in our MEFs by expressing MPS1 S820A, but these cells never entered mitosis probably because the SAC cannot be established in the absence of MPS1 activity. Nonetheless, Cdk1/cyclin B phosphorylation of MPS1 S820 may be a prerequisite for the kinetochore localization of MPS1 where it could acquire phosphorylation of other residues, resulting in enhanced activity. Of note, Mastl^NULL^ MEFs treated with microtubule poisons were transiently arrested with functional SAC signalling in early mitosis (NEBD). However, the SAC is prematurely silenced in Mastl^NULL^ MEFs. There are two possibilities; (1) Mastl does not regulate SAC and MPS1 during early mitosis, or (2) although MPS1 is hypophosphorylated and less active in Mastl^NULL^ MEFs, its remaining activity may be sufficient to initiate SAC signalling in early mitosis. The latter is in agreement with previous observations that 10% of MPS1 activity is sufficient to activate the SAC [33,37] by recruiting the Mad1-Mad2 complex to the kinetochores [29,37,38], which subsequently activates Mad2 to form the mitotic checkpoint complex (MCC) complex [39]. Our data indicate that maintenance of MPS1 activity by Mastl may be required to persistently localize Mad1 to the kinetochores, thereby sustaining SAC signalling.

We were able to detect three discernible phenotypes in Mastl^NULL^ MEFs; (i) delayed entry into mitosis possibly due to chromosome condensation defects, (ii) premature silencing of the SAC in the presence of chromosome segregation defects [this is the major focus of this manuscript], and (iii) cytokinesis defects mediated by the dephosphorylation of PRC1 [8]. While the phosphorylation status of MPS1 and the SAC are affected by Mastl deletion, this does not explain the entire phenotype observed in Mastl^NULL^ MEFs. MPS1 is likely not the only protein hypophosphorylated in the absence of Mastl. It is therefore evident that other targets are implicated in Mastl^NULL^ phenotype and that restoring MPS1 phosphorylation unlikely rescues the global defects due to Mastl loss. Nonetheless, our work has connected two important pathways, Greatwall kinase/Mastl->PP2A/B55 and Cdk1 in the regulation of the SAC, highlighting the importance of fine-tuning the signals through intricate feedback loops. Future studies will likely uncover additional mechanisms how SAC signalling is regulated in detail and new substrates of the Greatwall kinase/Mastl->PP2A/B55 pathway.

## Materials and Methods

### Ethics statement

All animal studies were approved by and have be conducted in a humane manner following the rules of the Biological Resource Centre (BRC) Institutional Animal Care and Use Committee (IACUC) of A*STAR at Biopolis, Singapore (IACUC protocol #140927).

### Generation of Mastl^FLOX^ mice and other transgenic lines used

The Mastl^FLOX^ mouse strain was generated as described previously [17].

Mastl conditional knockout mice were crossed with β-actin-Flpe transgenic mice [40] (strain name: B6.Cg-Tg(ACTFLPe) 9205Dym/J; stock no.: 005703; The Jackson Laboratory) to remove the neomycin cassette, which resulted in the Mastl^FLOX^ allele. The Mastl^NULL^ allele was then generated by crossing Mastl^FLOX^ mice with β-Actin-Cre transgenic mice [41] (strain name: FVB/N-Tg(ACTB-cre)2Mrt/J; stock no.: 003376; The Jackson Laboratory), which deletes exon 4 and causes a frame shift. Liver specific Mastl knockout was accomplished by crossing Mastl^FLOX^ mice with Albumin-Cre transgenic mice [42]. Tamoxifen inducible conditional knockouts were created by crossing with either Esr1 (CreERT2) [43] (strain name: B6.Cg-Tg(CAG-cre/Esr1*)5Amc/J; stock no.: 004682; The Jackson Laboratory) or Rosa26-CreERT2 mice [44]. To induce Mastl gene deletion in adult mice (8–10 weeks old), Mastl^+/FLOX^ or Mastl^FLOX/FLOX^ mice with two copies of the CreERT2 transgene (Rosa26-CreERT2^TG/TG^) were intraperitoneally injected with 1mg tamoxifen (Sigma-Aldrich, #T5648) dissolved in 50μl corn oil (Sigma-Aldrich, #C8267) for three consecutive days. For viability analysis, 4 adult mice of each genotype were used. After induction of recombination, Mastl^NULL/NULL^ mice died within 7–8 days while the Mastl^+/NULL^ mice remained viable with no visible phenotypical abnormalities for 4 months before they were euthanized. Mice were housed under standard conditions with food and water available ad libitum and maintained on a 12 hour light/dark cycle. Mice were fed a standard chow diet containing 6% crude fat.

### Isolation and culture of primary mouse embryonic fibroblasts

Primary mouse embryonic fibroblasts (MEF) of the Mastl^FLOX/FLOX^ Esr1 (CreERT2) genotype were isolated from E13.5 mouse embryos as described previously [18]. Briefly, the head and the visceral organs were removed, the embryonic tissue was chopped into fine pieces by a razor blade, trypsinized 15 minutes at 37°C, and finally tissue and cell clumps were dissociated by pipetting. Cells were plated in a 10cm culture dish (passage 0) and grown in DMEM (Invitrogen, #12701–017), supplemented with 10% fetal calf serum (Invitrogen, #26140) and 1% penicillin/streptomycin (Invitrogen, #15140–122). Primary MEFs were cultured in a humidified incubator with 5% CO_2_ and 3% O_2_.

### Cell cycle synchronization of MEFs

Primary and immortalized MEFs were synchronized at the G0/G1 phase of the cell cycle by culturing at high confluence (contact inhibition) and starvation in reduced serum containing growth media (0.2% fetal calf serum) for 72 hours. Recombination at the Mastl locus was induced only during the last 24 hours when majority of the cells had already arrested at the G0/G1 phase. In paired experiments comprising control and Mastl deficient conditions, identical MEF clones treated with DMSO (Control or Mastl^FLOX^) or 20 ng/ml 4-OHT (Mastl^NULL^) were used. To induce synchronized entry into cell cycle, cells were trypsinized and replated. Mitotic arrest was achieved by the addition of microtubule poisons and were performed between 20–24 or 24–28 hours after release for primary cells or immortalized MEFs, respectively, and the cells were subsequently processed and analyzed as decribed below.

### Proliferation assay and FACS analysis

For Alamar Blue proliferation assays; 1500 cells were plated in 96-well plates in 5 replicates, with or without prior 4-OHT (Sigma, #H7904) treatment to induce Mastl knockout. Starting from 24 hours after seeding, cells were incubated in 150 μl of assay medium (1:9 ratio of Alamar Blue (AbD Serotec, #BUF012B) to growth medium) for 4 hours and metabolic activity was quantified by measuring the fluorescence at 590nm.

For BrdU labeling and FACS analysis: MEFs were grown to confluence in 15 cm dishes and serum starved for 72 hours in 0.2% serum containing growth medium. To induce Mastl knockout, 4-OHT was added during entire starvation period (S2C Fig). To induce synchronized entry into cell cycle, cells were trypsinized and replated in 10cm dishes in full growth medium. To monitor S phase, cells were labeled with 100 μM BrdU (BD Pharmingen, #550891) for 1 hour before collection of the cells at different time points. At the end of each time point, cells were trypsinized and fixed in -20°C cold 70% ethanol, stained with APC conjugated anti-BrdU antibodies (BD Pharmingen, #623551) and propidium iodide (Sigma, #81845). Cell cycle analysis was performed using FACSCalibur flow cytometer (BD Biosciences) and resulting data were analyzed by FlowJo 8 software.

### Immortalized MEF lines, transfections, retroviral and lentiviral transductions

Primary Mastl^FLOX/FLOX^ MEFs were immortalized by serial passaging for 30 times using a modified 3T3 protocol [45]. To create cell lines where Mastl gene knockout can be induced by the addition of 4-OHT, cells were infected with pBABE-CreERT2 (PKB971) or pWZL-CreERT2 (PKB931) retroviral constructs and selected with 2.5 μg/ml puromycin or 10 μg/ml blasticidin, respectively. Immortalized cells displayed essentially the same phenotype as the primary MEFs after induction of the Mastl gene knockout. However after synchronization and release into full growth medium, they were delayed for about 4 hours to enter mitosis. For this reason, mitotic arrest studies in immortalized MEFs were performed between 24–28 hours after release, instead of 20–24 hours used for primary cells. Cell lines expressing H2B-YFP (PKB1515), mAg-hGeminin^1-110^ (PKB1572) and HA-hMPS1 (PKB1733) were created by lentiviral transduction of these immortalized MEFs.

MEFs were transfected in 10cm Petri dishes using X-tremeGene (Roche) with 5μg of plasmid encoding Myc-MPS1 (PKB1714) or Δ85cyclinB1 (PKB1851). 293FT cells were transfected by the calcium phosphate method during 8 hours with plasmids encoding HA-B55 (PKB1848) or HA-B56 (PKB1849) and cell extracts were collected after 48 hours.

### Antibodies and plasmids

Commercially available antibodies used for immunoblot or immunofluorescence microscopy staining are: rabbit anti-Cdk1 (Santa Cruz, #SC-954), mouse anti-Cdk2 (Santa Cruz, #SC-6248), rabbit anti-phospho Cdk1 Y15 (Cell Signalling #9111), rabbit anti-cyclin A2 (Santa Cruz, #SC-596), mouse anti-cyclin B1 (Cell Signaling, #4135), rabbit anti-phospho-Histone H3 Ser10 (Cell Signaling, #9701 or Upstate, #06–570), mouse anti MPS1-N terminal (EMD Millipore, #05–682), mouse anti-MPS1-CT, clone 4-112-3 (Millipore, #05–683), mouse anti-MPS1 (Sigma, #WH0007272M1), rabbit anti-MPS1 (Sigma, #HPA016834), rabbit anti-MPS1 (Santa Cruz, #SC-540), rabbit anti-CDK phospho substrates (Cell Signaling, #9477), mouse anti-HA tag (Cell Signaling, #2367), mouse anti-Myc (Clontech, #631208), mouse anti-Hsp90 (BD Transduction Labs, #610419), goat anti-actin (Santa Cruz, #SC-1616), human anti-Centromere antibodies (Antibodies Incorporated, #15–234), rabbit anti-Aurora B (Santa Cruz, #SC-25426), mouse anti-ENSA (Santa Cruz, #SC-81883), rabbi anti-PP2A A (Cell Signaling, #2039), rabbit anti-PP2A C (Sant Cruz, #SC-14020), and rabbit anti-Mad1 (Santa Cruz, #SC-222). Rabbit polyclonal antisera against Mastl were raised using an N-terminal 6-His tagged fragment from mouse Mastl (residues 461 to 694, PKB908) as antigen using a published protocol [46]. Phospho-specific MPS1 antibodies [24] have been described before and were kindly provided by Pat Eyers (University of Liverpool).

For FACS analysis of mitotic cells, Alexa 647 conjugated antibodies against phospho-Histone H3 Ser10 (Cell Signaling, #9716) were used. For histological analysis of mitotic cells, sections were stained with rabbit anti-phospho histone H3 (Ser10) antibodies (Upstate, #06–570).

The plasmid encoding Myc tagged human MPS1 (PKB1714) was described before [22]. The plasmid encoding mAg tagged human Geminin^1-110^ (PKB1572) was described before [47] and was kindly provided by Atsushi Miyawaki (RIKEN). The plasmid encoding non degradable Δ85cyclinB1 human (PKB1851) was described before [32]. The plasmids encoding HA-tagged B55α and B56α (PKB1848 and PKB1849) were kindly provided by David Virshup (Duke-NUS).

### Immunoblot, immunoprecipitations, kinase and phosphatase assays

Cells and tissues were lysed in EBN buffer (80 mM β-glycerophosphate pH 7.3, 20 mM EGTA, 15 mM MgCl_2_, 150 mM NaCl, 0.5% NP-40, 1 mM DTT, and protease inhibitors (20 μg/ml each of leupeptin, chymostatin, and pepstatin (Chemicon, EI8, EI6 and EI10)) for 20 min with constant shaking at 1200 rpm. Lysates were centrifuged for 30 min at 18,000g at 4°C and supernatants were snap frozen in liquid nitrogen and stored at -80°C. 10 μg of protein extracts were separated on 10% or 12.5% polyacrylamide gels, transferred onto polyvinylidene difluoride membranes (PVDF, Millipore, #IPVH0010) using a semi-dry system and blocked in tris-buffered saline (TBS) with 0.1% Tween20 and 4% non fat dry milk (Bio-Rad, #1706404). Blots were probed with the appropriate primary antibodies overnight at 4°C, followed by secondary goat anti-mouse (Pierce, #0031432) or anti-rabbit antibodies (Pierce, #0031462) conjugated to horseradish peroxidase and developed using enhanced chemiluminescence (PerkinElmer, #NEL105001EA).

Affinity purification/immunoprecipitations and Cdk/cyclin kinase assays were performed as described previously [46] with minor modifications. Briefly, 100–250 μg of protein extract were incubated with beads conjugated to Suc1 (Upstate, #14–132) or antibodies against Cdk1 (Santa Cruz, #SC-954AC), Cdk2 [46], cyclin A2 (Santa Cruz, #SC-751AC), cyclin B1 [46] overnight at 4°C in EBN buffer supplemented with 1 mg/ml ovalbumin (Sigma, #A5503). Antibodies were pre-coupled to protein A (Roche, #11719408001) or protein G (Roche, #11719416001) agarose beads. Following two washes in buffer EBN and one wash in buffer EB (EBN without NP-40), the precipitated proteins were used in kinase assays to determine the levels of kinase activity against the substrate histone H1 (Roche, #11004875001). Kinase assays were performed by incubating the immunoprecipitated proteins on beads in EB buffer with 10 mM DTT, 15 μM ATP, 5 μCi [ϒ-^32^P]ATP (PerkinElmer, #NEG502A) and 1.5 μg histone H1 for 30 min at room temperature. After inactivation with SDS-PAGE sample buffer, electrophoresis on polyacrylamide gel, fixation and staining in Bismarck Brown/Coomassie blue, quantification of incorporated radioactivity was performed with a phosphoimager (Fujifilm, FLA-7000).

For kinase assays to measure MPS1 activity, immortalized MEFs were used. Cells were synchronized and Mastl deletion was induced with 4-OHT. At the end of the serum starvation period, 2 million cells were seeded in 10 cm dishes in full growth medium while simultaneously being transfected with the Myc-MPS1 plasmid (PKB1714). 24 hours after seeding into full medium, cells were arrested in mitosis by the addition of 500 ng/ml nocodazole for 4 hours. Cells were collected and protein extracts were prepared as described above. The MPS1 kinase was immunoprecipitated from 1mg protein extracts by agarose beads conjugated to anti-Myc antibodies (Clontech, #631208). Kinase assays were performed in a 20μl reaction buffer (50 mM Tris-Cl pH7.5, 10 mM MgCl_2_, 100μM cold ATP, 5μCi [ϒ-^32^P]ATP, 1mM DTT, 4μM β-glycerophosphate, 1 mM EGTA) using 5μg of myelin basic protein (Sigma-Aldrich, #M1891) as substrate, for 30 minutes at room temperature. Kinase activity was detected as described above.

For kinase assays to test Cdk1/cyclin B1 kinase activity on MPS1 S820 residue, 5μg GST or GST-MPS1 peptide (residues 811–831; PKB1759 [S820S] and PKB1760 [S820A]) fusion protein bound to glutathione beads was used as substrates in a reaction volume of 20μl in a kinase reaction buffer comprised of 80 mM β-glycerophosphate pH 7.3, 20 mM EGTA, 15 mM MgCl_2_, 10 mM DTT, 100 μM cold ATP, 1 mM NaF and 5 μCi [ϒ-^32^P]ATP. 50 ng of purified Cdk1/cyclin B1 complexes (Cell Signaling, #7518) was added per reaction and incubated for 30 minutes at room temperature. Phosphorylation of GST-MPS1 fusion proteins was measured as above.

For phosphatase assay to test PP2A/B55 or PP2A/B56 activity on phosphorylated S820 residue, the previously ^32^P phosphorylated GST-MPS1 peptide fusion protein bound to gluthatione beads were washed twice in EB buffer (EBN without NP40) and twice in HEPES buffer (100 mM HEPES pH 8, 10 mM DTT, 10 mM MgCl_2_). Washed GST-MPS1 fusion protein was incubated in HEPES buffer complemented of protease inhibitors (20 μg/ml each of leupeptin, chymostatin, and pepstatin (Chemicon, EI8, EI6 and EI10)) for 4 hours at 30°C in the presence of immunoprecipitated HA-tagged B55 or B56 complexes from transfected 293FT cell extracts. The levels of phosphorylated GST-MPS1 was determined as above.

### Immunofluorescence and time-lapse video microscopy

MEF grown on coverglass-bottom chamber slides (Lab Tek) were fixed with 4% PFA or ice-cold MeOH. The fixed cells were permeabilized with 0.5% Triton X-100 and exposed to TBS containing 0.1% Triton X-100 and 2% BSA (AbDil). Images were acquired at RT with 3D-SIM using a Super Resolution Microscope (Nikon) equipped with an iXon EM+ 885 EMCCD camera (Andor) mounted on a Nikon Eclipse Ti-E inverted microscope with a CFI Apo TIRF (100x/1.40 oil) objective and processed with the NIS-Elements AR software. For time-lapse video microscopy, immortalized MEFs expressing the fluorescent fusion proteins (as described above) were synchronized by serum starvation. Cells were released in full growth medium and plated in coverglass bottom chamber slides (Nunc, #155380 and #155383). The images were captured every 10 min upon release using live-cell fluorescent microscopy with an iXon EM+ 885 EMCCD camera (Andor), a CFI Plan Fluor (20x/N.A. 0.45) objective and a Stage Top Incubation with Digital CO2 mixer (Tokai). The images were processed using NIS-Elements AR software.

### Primers, genotyping, RT-PCR, and qPCR

For PCR genotyping of Mastl wild type, FLOX, and NULL alleles, primers Pr1 (PKO860), Pr2 (PKO862), and Pr3 [PKO863] (S2 Table) were used at 1μM final concentration. Briefly, cells or tissue pieces to be genotyped were lysed by boiling in lysis solution (25mM NaOH pH 12, 0.2mM EDTA) for 20–30 minutes to extract genomic DNA [48]. Alkaline pH was neutralized by the addition of an equal volume of neutralization buffer (40mM Tris-HCl pH 5). 1 μl of the resultant genomic DNA solution was used as a template in a 20 μl volume of PCR reaction, using 0.5 units of MangoTaq polymerase (Bioline). 35 PCR cycles with 30 seconds denaturation at 94°C, 30 seconds annealing at 66°C, and 30 seconds extension at 72°C were performed to amplify different alleles of Mastl gene resulting in a band of 200bp (Mastl^WT^), 304bp (Mastl^FLOX^), or 500bp (Mastl^NULL^). Total RNA was extracted using MN NucleoSpin RNA II kit according the manufacturer’s protocol. For each RT-PCR reaction, first strand cDNA was synthesied from 1μg total RNA using the Maxima First Strand cDNA synthesis kit (Thermo Fisher, K1642). Mastl mRNA levels at different time points after 4-OHT induction of asynchronous primary MEFs were determined by RT-PCR using primers PKO1535 and PKO1536 (S2 Table). PCR amplification was carried out using the Maxima SYBR Green qPCR Master Mix (Fermentas, K0252) and the appropriate primer pair (see S2 Table). The reactions were monitored continuously in a Rotor-Gene thermal cycler (Corbett Research) using the following program: 95°C for 10 min, followed by 40 cycles of 95°C for 15 sec, 55°C for 30 sec, and 72°C for 30 sec. All data were normalized to the expression levels of eEF2 housekeeping gene using the (2^-ΔΔCt^) method.

### SILAC Mass Spectrometry (MS)

Primary Mastl^FLOX/FLOX^ Esr1 (CreERT2) MEFs were cultured in either media containing light isotopes of L-lysine-(^12^C_6_^14^N_2_) [K0] and L-arginine-(^12^C_6_^14^N_4_) [R0] or media containing stable heavy isotope L-lysine-(^13^C_6_^15^N_2_) [K8] and L-arginine-(^13^C_6_^15^N_4_) [R10] for three passages for a complete exchange of isotopes. Cells were synchronized by serum starvation and released to enter the cell cycle as usual. Cells were treated with 5μM Eg5 kinesin II inhibitor for 4 hours between 20–24 hours after serum release. Mitotic cells were isolated by pipetting and lysed in 8 M urea lysis buffer. Equal amounts of cell lysates from light (K0R0) and heavy (K8R10) cells were mixed. A total of two biological replicates were carried out, namely one Forward (light Mastl^FLOX^ MEFs versus heavy Mastl^NULL^ MEFs) and one Reverse (heavy Mastl^FLOX^ MEFs versus light Mastl^NULL^MEFs) experiments.

#### Preparation of peptide samples

Protein extracts in 8 M urea lysis buffer were reduced by the addition of 1/10 volume of 45mM DTT to the cleared cell supernatant and incubated for 20 min at 60°C. The samples were cooled to room temperature followed by addition of 110 mM iodoacetamide at a volume equal to the DTT solution and incubated for 15 min at room temperature in the dark. The samples were then diluted 4-fold to a final concentration of 2 M urea in 20 mM HEPES pH8.0. Sequencing-grade trypsin (Promega) was added with a trypsin to protein ratio of 1:50 (w/w). After overnight digestion at room temperature, the peptide solution was cleaned up using EmporeTM C18-SD Cartridge (3M Empore). The amount of peptide obtained was measured using Nanodrop (ThermoScientific).

#### Phosphopeptide enrichment

Phosphopeptides were enriched from the tryptic peptides as described with slight modifications [49]. Approximately 230 μg of in-solution digested tryptic peptides reconstituted in 40 mM ammonium bicarbonate were diluted 4 times with 1M glycolic acid, 80% acetonitrile (v/v), 2% TFA (v/v) and added to 1.25 mg of ‘Titansphere TiO_2_ 10μm’ (GL Sciences Inc., Japan). The mixture was incubated on a rotating wheel for 10 min at room temperature followed by centrifugation at 4000g for 3 seconds. The supernatant was collected and mixed with another portion of the beads and incubated as above. The bead-pellets from each incubation were separately transferred to a 200 μl pipet tip plugged with two layers of C8 empore disks (3M Empore). The beads were washed 3 times with 1M glycolic acid, 80% acetonitrile (v/v), 2% TFA (v/v) solution and 3 times with 80% acetonitrile (v/v), 0.1% TFA (v/v) solution. Finally, the phosphopeptides were eluted from the beads with 140 μl 40% acetonitrile (v/v) containing 15% NH_4_OH (m/v) and were vacuum-concentrated to ∼50 μl. The phosphopeptides were acidified and stored on a stage tip [50] before being analysed by LC-MS.

#### MS analysis

Each sample was analyzed in duplicate on an Orbitrap (Thermo Fisher). Survey full scan MS spectra (m/z 300–1400) were acquired with a resolution of r = 60,000 at m/z 400, an AGC target of 1e6, and a maximum injection time of 500 ms. The ten most intense peptide ions in each survey scan with an ion intensity of >2000 counts and a charge state ≥2 were isolated sequentially to a target value of 1e4 and fragmented in the linear ion trap by collisionally-induced dissociation using a normalized collision energy of 35%. A dynamic exclusion was applied using a maximum exclusion list of 500 with one repeat count, repeat, and exclusion duration of 30 s.

#### MS data analysis

MS data were processed using MaxQuant (Version 1.3.0.5) [51,52] against uniprot 2014–01 mouse database containing 262 commonly observed contaminants. Database searches were performed with tryptic specificity allowing maximum two missed cleavages and two labeled amino acids as well as an initial mass tolerance of 6 ppm for precursor ions and 0.5 Da for fragment ions. Cysteine carbamidomethylation was searched as a fixed modification, and N-acetylation, oxidized methionine, phosphorylated serine/threonine/tyrosine were searched as variable modifications. Labeled arginine and lysine were specified as fixed or variable modifications, depending on the prior knowledge about the parent ion. SILAC peptide and protein quantification was performed using default settings. Maximum false discovery rates were set to 0.01 for both protein and peptide. Proteins were considered identified when supported by at least one unique peptide with a minimum length of six amino acids. For the complete and simplified dataset see S3 and S4 Tables. The mass spectrometry proteomics data have been deposited to the ProteomeXchange Consortium via the PRIDE [53] partner repository with the dataset identifier PXD004882.

### Statistical analysis

To test the statistical significance of the distribution of samples displayed in Fig 2D a Student’s t-test applett available at http://www.math.kent.edu/~blewis/stat/tTest.html website was used. For Figs 4B–4D, 5B and 5C and S9B and S9C Fig, Student’s t-test was directly determined in the program Prism.

## Supporting Information

S1 Fig Generation and analysis of Mastl conditional knockout mice. (A) To generate Mastl knockouts, Mastl^FLOX^ mice were crossed with ß-actin-Cre mice expressing Cre recombinase ubiquitously in all tissues including germ line. The resulting Mastl^WT/NULL^ mice were interbred and the genotypes of the offspring were analysed at weaning (P21) and embryonic days 13.5, 10.5 (midgestation), 7.5 (decidua), and 3.5 (blastocyst). Asterisk indicates that all the Mastl^NULL/NULL^ embryos analysed were significantly smaller in size compared to wild type (WT) Mastl^WT/WT^ (see C and D). (B) H&E staining of histological sections from control Mastl^WT/WT^ and Mastl^NULL/NULL^ embryos at E7.5. Scale bar 200μm. (C) Control Mastl^WT/WT^, Mastl^WT/NULL^, and knockout Mastl^NULL/NULL^ embryos at E7.5 stage were isolated and photographed. Scale bar 500μm. (D) To generate control (Mastl^WT/WT^ or Mastl^WT/NULL^) and Mastl^NULL/NULL^ embryos, male mice with Mastl^FLOX/FLOX^Rosa26^CreERT2/CreERT2^ genotype were bred with female mice with Mastl^WT/FLOX^Rosa26^WT/WT^ genotype. Pregnant females were injected intraperitoneally with 1mg tamoxifen dissolved in 50μl corn oil for three consecutive days starting from E10.5. Embryos were collected at E13.5 and genotyped for recombination. Histological sections from Mastl^WT/NULL^ and Mastl^NULL/NULL^ embryos were stained with H&E. Mastl deficient embryos (ii and iv) displayed reduced size, haemorrhaging, and reduced cell proliferation with nuclear morphology abnormalities. (E) (i-ii) Liver sections from 10-week old control Mastl^WT/NULL^ [Mastl^WT/FLOX^Alb-Cre; (i)] and liver specific knockout Mastl^NULL/NULL^ [Mastl^FLOX/FLOX^Alb-Cre; (ii)] were analysed by H&E staining. Mastl deficient hepatocytes (ii) displayed abnormalities in nuclear morphology with reduced cell density throughout the liver. Scale bar 50μm. (iii-iv) 8–10 week-old mice were injected with tamoxifen to induced Mastl gene deletion in the entire body as described in Methods section. 96 hours after the first injection, mice were sacrificed and the intestinal tissue was histologically analysed by H&E staining. Mastl^NULL/NULL^ mice (Mastl^FLOX/FLOX^ Rosa26^CreERT2/CreERT2^) displayed severe degeneration of the crypt morphology with decreased cellularity and aberrant nuclear morphologies in the microvilli (iv). Control mice Mastl^WT/NULL^ (Mastl^WT/FLOX^Rosa26 ^CreERT2/CreERT2^) had a normal intestinal morphology (iii). Scale bar 50μm.
(PSD)

S2 Fig Analysis of Mastl^NULL^ MEFs. (A) Freshly isolated primary MEFs of the Mastl^FLOX/FLOX^Esr1 (CreERT2) genotype were induced to undergo recombination in the Mastl locus by the addition of 20ng/ml 4-hydroxtamoxifen (4-OHT) to the culture medium. Cells were collected at the indicated time points after induction of recombination and RNA and protein extracts were prepared. Loss of Mastl gene expression at RNA and protein level was analysed by RT-PCR and immunoblotting, respectively. (B) MEFs as in A were grown for 48 hours in culture medium containing DMSO or 4-OHT prior to fixation and analysis of Mastl expression by immunofluorescence staining using antibodies against Mastl. Mastl^NULL^ MEFs ceased to proliferate and displayed abnormalities in nuclear morphology with frequent anaphase bridges (see also Fig 1A, 1D and 1E). Bright-field phase-contrast microscopy images indicated a senescent morphology of the cells. Scale bars 100 μm (left and middle panels) and 250 μm (right panels). (C) Primary MEFs as in A were synchronized by serum starvation for 72 hours while Mastl deletion was simultaneously induced by the addition of 4-OHT to the starvation medium. Cell cycle re-entry was initiated by plating the cells in complete medium at reduced cell density. Cells were pulse labelled with BrdU as an indicator of S phase and collected at the indicated time points. Mastl deficient cells were arrested with an increased proportion of cells in the G2/M phase and became increasingly polyploidy after continued culturing in full growth medium.
(PSD)

S3 Fig Expression of cell cycle regulators and kinase assays in Mastl deficient MEFs. (A) Primary MEFs as in S2 Fig, were synchronized by arresting in G0/G1 phase of the cell cycle by 72 hours serum starvation while Mastl deletion was induced only during the last 24 hours of starvation period after majority of the cells had already been arrested. Cells were released to enter cell cycle and collected at different time points for preparation of protein extracts. Cdk1^FLOX/FLOX^ Esr1 (CreERT2) MEFs were treated similarly and collected 48 hours after release. 10μg of the protein extracts were separated with SDS-PAGE and analyzed by immunoblot using the indicated antibodies. (B) Cdk/cyclin complexes were immunoprecipitated from the protein extracts prepared as in A, using beads conjugated with the indicated antibodies. Kinase assays were performed using histone H1 as a substrate and phosphorylated H1 was separated by SDS-PAGE and analysed by phosphoimager. (C) Quantification of histone H1 phosphorylation in B. Histograms for different time points were normalized to the first sample (Control, 12 hours) in the same chart. NIU, normalized intensity units.
(PSD)

S4 Fig Increased mitotic index and anaphase bridges in Mastl^NULL^ hepatocytes after partial hepatectomy. (A) Mastl^WT/FLOX^ or Mastl^FLOX/FLOX^ mice carrying Rosa26-CreERT2 transgene were injected with 1mg tamoxifen for two consecutive days to induce recombination mediated Mastl^NULL^. 48 hours after first injection, 70% of the liver was removed by partial hepatectomy (PHx). Mice were sacrificed 48 hours after PHx and liver tissue was analyzed as below. H&E stained histological sections from control and Mastl^NULL^ liver before and 48 hours after PHx are shown. Yellow arrows indicate nuclei with anaphase bridges in Mastl^NULL^ hepatocytes. (B) 48 hours after PHx, the liver was isolated and sections were stained using anti-phospho-histone H3 (Ser10) antibodies, to monitor cells entering mitosis. Red arrows denote metaphase/anaphase cells while the black arrows denote prophase cells with intact nuclei. (C) Feulgen staining of sections in (B). Red arrows indicate nuclei with anaphase bridges. (D) Wild type liver protein extracts as previously described in [18], were analyzed for the expression of Mastl after PHx by immunoblot using the indicated antibodies. Expression of Mastl is induced after PHx. (E) Control and Mastl^NULL^ liver extracts were analyzed before and 48 hours after PHx. Expression of cell cycle genes Cdk1, cyclin A2, and Mastl are increased after PHx. Mastl^NULL^ liver (lanes 7 and 8) do not show an increased expression of Mastl compared to controls Mastl^WT/WT^(lanes 5 and 6) as expected.
(PSD)

S5 Fig Mitotic slippage in Mastl^NULL^ MEFs. (A) FACS charts of the cells treated as in Fig 2E. Mitotic slippage was determined by decreases in the number of cells positive for phospho-histone H3 (pH3) staining after nocodazole treatment. (B-C) Primary MEFs as in Fig 2E were synchronously released to enter the cell cycle, were treated with 500ng/ml of nocodazole starting from 20 hours. Cells were collected by pipetting (mitotic shake-off), further incubated in the presence of nocodazole and fixed at different time points to determine the percentage of positive cells for the phospho-histone H3 (pH3) on Ser10 by FACS analysis. For (C), cells were treated further with 200nM OKA while for (B), Mastl^FLOX^ MEFs were treated further with DMSO (black bars) or 10nM of reversine (blue bars).
(PSD)

S6 Fig Immunoblot for MPS1 kinase assays. (A) 10μg of the protein extracts and 20% of the immunoprecipitated MPS1 that were used in kinase assays as described in Fig 4B, were separated by SDS-PAGE and immunoblotted using the indicated antibodies to verify the protein depletion of Mastl and the equal levels of immunoprecipitated MPS1. (B) GST-MPS1 peptide fusion protein was purified and subjected to *in vitro* kinase assay with immunoprecipitated HA-Mastl in absence (-, left) or presence (+, right) of recombinant Cdk1/cyclin B1 complexes (see Material and Methods). Increased amount of whole cell extracts from HA-Mastl overexpressing 293T cells (0, 30, 75, 100μg), were immunoprecipitated with HA-beads and incubated in presence of GST-MPS1 peptide protein fusion. Western blot for HA-Mastl is shown in the top panel, Coomassie stained gel in the second panel from the top, phosphorylated GST-MPS1 peptide fusion protein short and long exposure in the bottom two panels, respectively. (C) Immortalized MEFs synchronously released into full growth medium were transfected with plasmids encoding non-degradable EGFP-Δ85cyclin B1 or EGFP together with Myc-MPS1. Myc-MPS1 was immunoprecipitated using anti-Myc antibodies from cells extracts prepared at 28 hours after release and subjected to *in vitro* kinase assay as in Fig 3E. ^32^P phosphorylated MBP signal intensities were normalized to the expressed levels of EGFP-Δ85cyclinB1/EGFP. The quantified results are shown in a bar graph for Mastl^FLOX^ and Mastl^NULL^. AU, arbitrary unit. (D) GST-tagged recombinant Rb protein was purified and subjected to *in vitro* kinase assay with recombinant Cdk1/cyclin B1 complexes (D; see Material and Methods). Phosphorylated GST-Rb protein was subjected to *in vitro* phosphatase assay using immunoprecipitated HA-tagged B55 or B56 complexes (upper panel). Signal intensities are shown as percentage compared to lane 2. Western blot for the PP2A A scaffold subunit is shown in the bottom panel.
(PSD)

S7 Fig mRNA expression profile for ENSA and the isoforms of PP2A regulatory subunitB55 and B56 in Mastl deficient MEFs. Primary Mastl^FLOX^ and Mastl^NULL^ MEFs as in S3A Fig were collected at different time points after release and total RNA were extracted to determine mRNA expression of ENSA, PPP2R2/B55 and PPP2R5/B56 isoforms by qPCR. Relative levels of mRNA were obtained using the comparative threshold method (2^-ΔΔCt^) relative to Mastl^FLOX^ MEFs at 12 hours.
(PSD)

S8 Fig Localization of phosphorylated MPS1 on T675 and T685 in late mitosis. Primary MEFs were synchronized and released to enter the cell cycle as in Fig 2F. Cells were treated with 500ng/ml nocodazole for 4 hours between hours 20–24 after serum release. Where indicated, cells were treated with 200nM OKA for one hour to inhibit PP2A activity between hours 23–24 after serum release. Cells were fixed with PFA or ice-cold MeOH and analysed by immunofluorescence microscopy using ACA and antibodies against MPS1-pST675 or -pT685. Late mitotic cells with a highly condensed chromosome mass typically caused by nocodazole treatment were selected for analysis. Insets represent the boxed areas. Scale bar 5 μm.
(PSD)

S9 Fig Localization of phosphorylated MPS1 on T675 and T685 in early mitosis. (A) Primary Mastl^FLOX^ and Mastl^NULL^ MEFs were treated as in Fig 4 to arrest in mitosis and were fixed. Immunofluorescence analysis was performed using ACA and antibodies against MPS1-pST685 or -pS820. Early mitotic phase was determined with cells containing lightly condensed chromosomes that were scattered in the cytoplasm just after NEBD. Insets represent the boxed areas. Scale bar 5 μm. (B, C) Quantification of kinetochore-localized MPS1-pT685 (B) and -pS820 (C) in nocodazole-arrested Mastl^FLOX^ and Mastl^NULL^ MEFs in early mitosis (N>10 cells per each condition with several kinetochores analyzed per cell, +/- standard deviation). To determine the statistical significance, a Student’s *t*-test was performed. (n.s. not significance; ***p<0.0001, Student’s *t*-test, unpaired).
(PSD)

S1 Table Identification of MPS1 as one of the substrates changing phosphorylation status. (PDF)

S2 Table Primers used in this study. (PDF)

S3 Table Complete phospho-proteomic mass spectrometry data. (XLSX)

S4 Table Simplified phospho-proteomic mass spectrometry data. (XLSX)

S1 Movie Mitotic exit in primary Mastl^NULL^ MEFs. (AVI)

S2 Movie Mitotic exit in immortalized Mastl^NULL^ MEFs. (AVI)
