# Supplementary material for: Loss of the Greatwall Kinase Weakens the Spindle Assembly Checkpoint
Source: PLoS Genet. 2016 Sep 15;12(9):e1006310. doi: 10.1371/journal.pgen.1006310 (PMC5025047; doi:10.1371/journal.pgen.1006310)
Supplement: S1 Table — (PDF) [file pgen.1006310.s010.pdf]

Induced/Mastl KO (K0R0)

Forward (Fwd): Control/Mastl WT (K0R0) + Induced/Mastl KO (K8R10)

7-Mar-14

Quantification software: MaxQuant v1.3.0.5

Search parameters: precursor tolerance - 6 ppm, MS/MS tolerance - 0.5 Da

Modifications: Acetyl (Protein N-term), Oxidation (M), Arginine-13C615N4 (R-full), Lysine-13C615N2 (K-full), phospho(STY)

Database: MOUSE.fasta (Jan,2014)

Min. Ratio Count: 1

Proteins	Positions within proteins	Gene names	Localization prob Rev	Score Rev	Localization prob Fwd	Score Fwd	Modified sequence	Ratio H/L normalized Rev	Ratio H/L normalized Rev___1	Ratio H/L normalized Rev___2	Ratio H/L normalized Rev___3	Ratio H/L count Rev	Ratio H/L normalized Fwd	Ratio H/L normalized Fwd___1	Ratio H/L normalized Fwd___2	Ratio H/L normalized Fwd___3	Ratio H/L count Fwd	Contaminant
Q99K01-	652	Pdxdc1	1	149.07	0.99996	108.64	_QIPVVGSVLNWFS(ph)PVQASQK_	6.4022	6.4022	NaN	NaN	2	0.11253	0.11253	NaN	NaN	4	
D3Z3Q3;	532;78	Smtn	0.9823	58.123	0.52336	38.736	_LGSVTHVTTFSHAS(ph)PGNR_	4.1538	4.1538	NaN	NaN	2	0.32055	0.32055	NaN	NaN	1	
Q99K48	452	Nono	0.99901	52.721	0.99995	57.629	_FGQAATM(ox)EGIGAIGGT(ph)PPAFNRPAPGAEFAPNKR_	3.772	3.772	NaN	NaN	4	0.25427	0.25427	NaN	NaN	4	
P26645	160	Marcks	0.99734	99.011	0.98009	79.089	_SFKLS(ph)GFSFK_	3.122	3.122	NaN	NaN	2	0.36731	0.36731	NaN	NaN	1	
E9Q616	3137	Ahnak	0.81949	102.52	0.83852	88.706	_FKMPFLS(ph)ISSPK_	2.9098	2.9098	NaN	NaN	1	0.37834	0.37834	NaN	NaN	1	
P35761;P	820;794	Ttk/MPS1	0.7862	82.36	0.80424	49.528	_YVLGQLVGLNS(ph)PNSILK_	2.6738	2.6738	NaN	NaN	1	0.39392	0.39392	NaN	NaN	1	
Q6NZJ6;	1096;1090;108	Eif4g1			0.99987	56.156	_ITKPGSIDSNNQLFAPGGRLS(ph)WGK_	2.6336	2.6336	NaN	NaN	2	0.53584	0.53584	NaN	NaN	4	
P99027	17	Rplp2	0.99966	243.24	0.96568	195.13	_YVASYLLAALGGNSS(ph)PSAK_	2.5521	2.5521	NaN	NaN	3	0.41527	0.41527	NaN	NaN	3	
P20152	144	Vim	1	66.809	1	45.992	_ILLAELEQLKGQGKS(ph)R_	2.0562	2.0562	NaN	NaN	3	0.61511	0.61511	NaN	NaN	2	
Q62523	272	Zyx	0.94304	63.488	0.8433	56.9	_FTPVVSKFS(ph)PGAPSGPGPQPNQK_	1.927	1.927	NaN	NaN	1	0.51013	0.51013	NaN	NaN	2	
Q8C156	83	Ncaph	0.99983	130.21	0.98102	70.529	_VFDLQFSTDSIHLAS(ph)PNR_	1.7863	1.7863	NaN	NaN	2	0.6088	0.6088	NaN	NaN	1	
D3Z576;(	2599;2566	Flnc			0.51141	39.737	_VTGPRLS(ph)GGHSLHETSTVLVETVTK_	2.4593	2.4593	NaN	NaN	1	0.66324	0.66324	NaN	NaN	1	
Q8BTM8	1084;1060	Flna	1	58.831	1	47.68	_AFGPGLQGGNAGS(ph)PAR_	2.3843	2.3843	NaN	NaN	2	0.49626	0.49626	NaN	NaN	1	
Q8BTM8	1750;1742;33	Flna	1	155.17	0.95781	34.437	_FGGEHVPNSPFQVTALAGDQPTVQT(ph)PLR_	1.77	1.77	NaN	NaN	2	0.56892	0.56892	NaN	NaN	1	
