# Supplementary material for: Loss of the Greatwall Kinase Weakens the Spindle Assembly Checkpoint
Source: PLoS Genet. 2016 Sep 15;12(9):e1006310. doi: 10.1371/journal.pgen.1006310 (PMC5025047; doi:10.1371/journal.pgen.1006310)
Supplement: S2 Table — (PDF) [file pgen.1006310.s011.pdf]

Purpose	Primer Name	Sequence
Genotyping	Pr1 (PKO860)	CATGCCTTCCTTGAAAGAGGTGGAC
Genotyping	Pr2 (PKO862)	GTGGGAGGAATTACAAGAGACAAC
Genotyping	Pr3 (PKO863)	GGCAGGTGGAGGCAAGAGCTCACAGA
5’ Probe	PKO719	TTAAGGAATCTGACCATTAACTG
5’ Probe	PKO720	GCCTTGTAAATTCACCTTGGTGCA
3’ Probe	PKO756	CCTAGTTGTAAAACGTAATGCTTA
3’ Probe	PKO757	AGGGATCCCTGTCCCTATCTTTA
qPCR Mastl fwd	PKO1535	GGAGTATCTTATTGGTGGAGA
qPCR Mastl rev	PKO1536	AGCATATTGTCCGGTTTCAA
Cloning GST-Mps1	PKO3471	CCTGGGATCCTATGTTCTGGGCCAA
Cloning GST-Mps1	PKO3472	GCCGCTCGAGTCAAGTTTTAGCAGCTTT
S820A mutagenesis	PKO3451	CAACTTGTTGGTCTGAATGCTCCTAACTCCATTTTGAA
S820A mutagenesis	PKO3452	TTCAAAATGGAGTTAGGAGCATTCAGACCAACAAGTTG
qPCR ENSA fwd	PKO3097	TAAAGGCCAAATACCCAAGC
qPCR ENSA rev	PKO3098	TTTGGCCATGTTGTAGTCTC
qPCR PPP2R2A/B55 alpha set A fwd	PKO4906	AGCCATAGCGGTCGTTATATG
qPCR PPP2R2A/B55 alpha set A rev	PKO4907	GCAGTCGTTTTCATACAGTGAG
qPCR PPP2R2A/B55 alpha set B fwd	PKO4908	GGAGGGAATGATATTCAGTGGT
qPCR PPP2R2A/B55 alpha set B rev	PKO4909	CAACTCTCCCACCTTTATCTCC
qPCR PPP2R2D/B55 delta set A fwd	PKO4918	TCCTTGTATGAGAACGACTGC
qPCR PPP2R2D/B55 delta set A rev	PKO4919	TGAGGCTTCCAGTGTAACATC
qPCR PPP2R2D/B55 delta set B fwd	PKO4920	TGCAACAGGAGACAAAGGTG
qPCR PPP2R2D/B55 delta set B rev	PKO4921	GGCTCATGACTCTGAAAGGTAC
qPCR PPP2R5A/B56 alpha set A fwd	PKO4922	TCCTACAAAGCCGAAAGACAG
qPCR PPP2R5A/B56 alpha set A rev	PKO4923	CTGCGAATACTGTGCATGTTG
qPCR PPP2R5A/B56 alpha set B fwd	PKO4924	TGGTGTAATTGTTGAATCAGCG
qPCR PPP2R5A/B56 alpha set B rev	PKO4925	AACTGTATGTGAGGCCAAGAG
qPCR PPP2R5B/B56 beta set A fwd	PKO4926	CTTCTCAGTTCCGCTATCAGAG
qPCR PPP2R5B/B56 beta set A rev	PKO4927	GTCAAACATCACCCCACATTG
qPCR PPP2R5B/B56 beta set B fwd	PKO4928	TTGACAGAGCATGTTATCCGG
qPCR PPP2R5B/B56 beta set B rev	PKO4929	CCTGGATCTTCACAAACTGGG
qPCR PPP2R5C/B56 gamma set A fwd	PKO4930	GCCAAATGTGTTTCCAGCC
qPCR PPP2R5C/B56 gamma set A rev	PKO4931	TTGAGTTGCGGTATAAGGACG
qPCR PPP2R5C/B56 gamma set B fwd	PKO4932	TTCCGTCCTTATACCGCAAC
qPCR PPP2R5C/B56 gamma set B rev	PKO4933	GCTTCTCTTTGAGTTTCTCTGC
qPCR PPP2R5D/B56 delta set A fwd	PKO4934	GGCCGAGATGTCCTATAAACTG
qPCR PPP2R5D/B56 delta set A rev	PKO4935	GGATGATGGTGACTGAGACTG

qPCR PPP2R5D/B56 delta set B fwd	PKO4936	AGGAACTCCAAAAGCCACTG
qPCR PPP2R5D/B56 delta set B rev	PKO4937	TCCTTCATTCGGAACCTGC
qPCR PPP2R5E/B56 epsilon set A fwd	PKO4938	TCTTGGATGTGATTGAACCGTC
qPCR PPP2R5E/B56 epsilon set A rev	PKO4939	AATAGAGTGCCCTTTCTGCC
qPCR PPP2R5E/B56 epsilon set B fwd	PKO4940	CATGGCCCCACTTACAGC
qPCR PPP2R5E/B56 epsilon set B rev	PKO4941	AGGGTCTTCACTGTCAAATAGC
qPCR eEF2 fwd	PKO3215	CACTTACCATCCCCCGTCAC
qPCR eEF2 rev	PKO3216	CTTTGGGGTCGCAGCTCTTA
